# Spatial and Temporal Characteristics of Normal and Perturbed Vesicle Transport

**DOI:** 10.1371/journal.pone.0097237

**Published:** 2014-05-30

**Authors:** Gary J. Iacobucci, Noura Abdel Rahman, Aida Andrades Valtueña, Tapan Kumar Nayak, Shermali Gunawardena

**Affiliations:** 1 Department of Biological Sciences, The State University of New York at Buffalo, Buffalo, New York, United States of America; 2 Department of Physiology and Biophysics, The State University of New York at Buffalo, Buffalo, New York, United States of America; Stanford University School of Medicine, United States of America

## Abstract

Efficient intracellular transport is essential for healthy cellular function and structural integrity, and problems in this pathway can lead to neuronal cell death and disease. To spatially and temporally evaluate how transport defects are initiated, we adapted a primary neuronal culture system from *Drosophila* larval brains to visualize the movement dynamics of several cargos/organelles along a 90 micron axonal neurite over time. All six vesicles/organelles imaged showed robust bi-directional motility at both day 1 and day 2. Reduction of motor proteins decreased the movement of vesicles/organelles with increased numbers of neurite blocks. Neuronal growth was also perturbed with reduction of motor proteins. Strikingly, we found that all blockages were not fixed, permanent blocks that impeded transport of vesicles as previously thought, but that some blocks were dynamic clusters of vesicles that resolved over time. Taken together, our findings suggest that non-resolving blocks may likely initiate deleterious pathways leading to death and degeneration, while resolving blocks may be benign. Therefore evaluating the spatial and temporal characteristics of vesicle transport has important implications for our understanding of how transport defects can affect other pathways to initiate death and degeneration.

## Introduction

Within axons the intracellular transport of vesicles, organelles, and biomolecules is essential for the maintenance of its structure and for its function. Such a complex pathway utilizes motor proteins, kinesin-1 and dynein, for anterograde and retrograde transport on a vast network of microtubules (MTs) [1]–[3]. Dysfunctions in this pathway can be detrimental to the cell’s structural integrity and its overall ability to function [4]. Recently, many vesicle classes have been identified that are transported within axons [5]–[7], but their spatial and temporal motility kinetics remain elusive, mainly due to the lack of a suitable model system for such analysis.

Although single particle motility analysis has not yet been accomplished within an entire mammalian organism, such analysis has been performed in filleted living *Drosophila* larvae under physiological conditions [8]. While methods for *in vivo* imaging within filleted larvae have been developed [8]–[11], imaging in intact larvae prompted the use of anesthetics due to the need for a completely immobile larva during imaging [12]–[14]. This restricted the length of imaging intervals and prevented continuous imaging because the larvae only survived short doses of anesthetics [15]–[17]. In addition, it is known that repeated anesthesia can inhibit neuronal physiology and alter neuronal processes such as vesicle transport [16], [17]. To circumvent these problems microfluidic devices have recently been used as a noninvasive approach to image cellular processes by capitalizing on the transparency of the larval cuticle [18]. However, larvae in microfluidic chambers only survived for a maximum of 12 hours, thus preventing the continuous imaging of cellular processes [19]. Moreover, despite the significant reduction in mobility, subtle larval locomotion movements were still observed, thus skewing vesicle analysis. Further, the high resolution imaging that is needed for quantifying the precise dynamics of single vesicle motility trajectories were not achieved in intact animals due to light scattering effects from the larval cuticle, rendering such analysis only sufficient for detecting general population shifts in vesicle trajectories as well as gross morphological changes in development [14], [19]. In contrast, primary neuronal cultures allow for continuous high resolution observation of vesicle motility, long-term, without the confounding effects of anesthetics and larval movements during recordings. Such analysis can lead to new insights on both the spatial and temporal mechanisms of vesicle motility and their role for neuronal growth and maintenance, which are currently unclear.

Here, we characterize both the spatial and temporal aspects of six vesicles/organelle motilities using a primary neuronal culture system by assessing a battery of movement parameters in an axonal neurite as they travel within a 90 micron field of view at a spatial resolution of 0.126 micron/pixel and a temporal resolution of 0.2 sec/frame over two days. We found striking temporal changes in vesicle/organelle transport indicating that these attributes are likely due to functional activities of the growing neuron. Reduction of kinesin-1 or dynein perturbed vesicle transport both spatially and temporally, and these affects likely contributed to the defects seen in neuronal growth. Further analysis indicated that not all blockages in an axonal neurite were static as previously thought but some were dynamic and could resolve over time. Our observations demonstrate the temporal behavior of blockages, and provide new insights into the mechanisms underlying vesicle transport and its role in the initiation of deleterious pathways causing cell death.

## Results

### Functional and Morphological Characteristics of Primary Neurons from *Drosophila* Larval Brains

**Figure 1:**
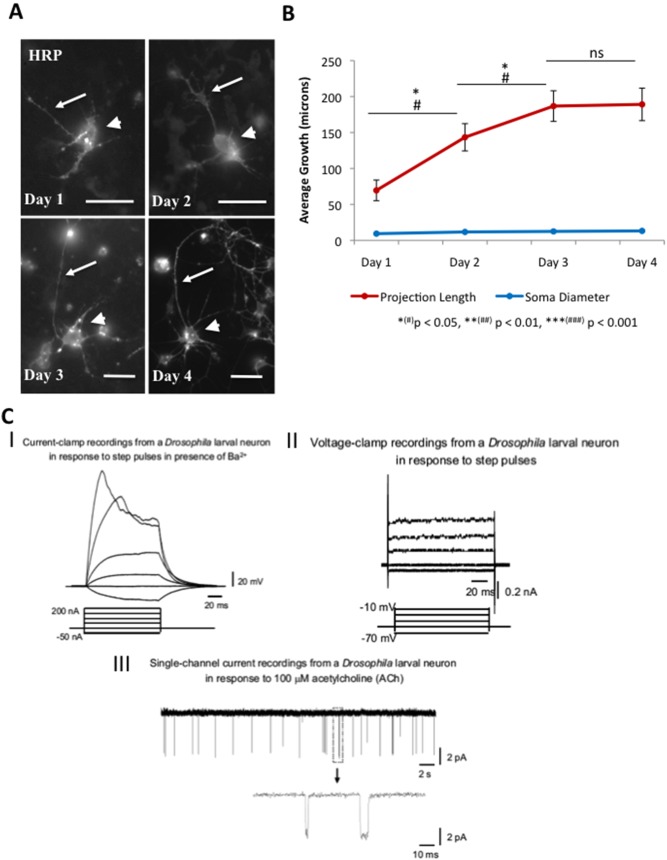
Primary Drosophila larval neurons are healthy and functional. Under our conditions, primary neurons generated from larval brains grew and retained their functional activity over several days. (**A**) Significantly longer neuronal projections were observed at day 2, day 3 and day 4 as assayed by both phase contrast (data not shown) and HRP staining. Neurons at day 1 and 2 were imaged at 100X, while neurons at day 3 and 4 were imaged at 60X. Cell body  =  arrowhead, neurite projection  =  arrow. Bar = 10 microns (**B**) Quantification of neuronal projection lengths at day 1 to day 3 showed significant growth increases (day 1 to day 2 p = 0.016, day 2 to day 3 p = 0.042), while growth plateau from day 3 to day 4. Note that no significant changes were seen in the growth of the cell body over day 1 to 4. (**C**) Electrophysiological recordings indicated that our primary neurons were functional. (I) Current clamp recordings using 50 nA step pulses indicate depolarizing voltage changes in whole cell membrane potential. Negative current generated hyperpolarizing membrane potential recordings confirm the presence of functional ion channels on membranes. (II) Voltage clamp recordings from a neuron in response to step pulses is shown. Both outward and inward currents were recorded revealing the presence of functional channels on the cell membrane. (III) Single-channel current recordings from a neuron in response to 100 µM Ach revealed characteristic acetylcholine channel activity. Periodic opening of these channels generated current peaks of about 6 pA. N = 10 cells *(#) p<0.05, **(##) p<0.01, ***(###) p<0.001 by two-tailed Student t-test (*) and by Bonferroni’s test (#). NS = not significant.

We adapted a protocol to generate primary neurons from larval brains [20] to visualize both the spatial and temporal aspects of vesicle motility in axonal neurites. Primary neurons grew robustly and survived for at least 5 days with no additional treatment of trophic factors [21]. We first quantitatively analyzed the growth of neurons by measuring the total length of the longest neurite projection as well as the diameter of the cell body, over 4 days in ten randomly selected neurons from more than 5 independently grown primary neuronal cultures generated from 3^rd^ instar larval brains. While no change was seen in the average growth of the cell body diameter, neuronal projection lengths showed significant changes from day 1 to day 3 indicating growth (Figure 1AB; Table S1). To further examine how expression of a vesicle protein affected the growth rate we evaluated GFP-tagged atrial natriuretic factor (ANF-GFP) expressing neurons over 4 days and compared these to non-GFP/YFP expressing neurons. While neurites expressing ANF-GFP significantly grew during day 1 to day 4 (Figure S1), no significant changes in growth was seen between neurites and cell bodies expressing ANF-GFP and neurites and cell bodies not expressing a fluorescent protein, indicating that expression of proteins do not significantly perturb growth in our cultures.

Early research demonstrated that most neurons harvested from *Drosophila* larval CNS developed neuronal processes with extensive branching and occasionally made contacts with other neurons within 24 hrs [22]. By 24 hrs, a single long neurite representing 80% of the cell’s total neurite length was seen [20]. Because we sought to restrict our analysis to axonal neurites, we assessed neurite differentiation in our culture system using two methods, 1) by using an antibody against Futsch, a MT associated protein homologous to MAP1B that is essential for synaptic growth and is enriched in the axonal cytoskeleton [23], [24], and 2) by evaluating MT polarity using EB1-YFP [11]. End binding protein 1 (EB1) is an evolutionarily conserved protein that localizes to the plus ends of growing MTs [25]. Neuronal cultures at day 1 and day 2 were fixed and stained with Futsch antibody. At both day 1 and day 2, Futsch was enriched to a single long neurite (Figure S2A). MT polarity was evaluated by assessing EB1-YFP to identify axonal and dendritic neurites. Quantification analysis done blind using a custom particle tracking program showed that at both day 1 and day 2, EB1-YFP was uni-directional in the longest neurite (Figure S2B; Movie S1; Movie S2), while bi-directional EB1-YFP tracks were observed in smaller neuritis, consistent with multi polar MT orientations [25] (Figure S2B; Movie S1; Movie S2). Taken together these observations indicate that our primary neurons show differentiation and are polarized at both day 1 and day 2.

A majority of previous cell culture studies in *Drosophila* have focused on the use of embryos [26]–[29], with only a few studies using third instar larvae for cultures [22], [30] it was necessary for us to first confirm that our cultured neurons from larval brains also maintained their normal physiological properties at later time points before using these cultures for transport analysis. Therefore we evaluated the functionality of our primary neurons to ensure that the neurons produced in our culture system in which motility analysis was to be performed were healthy by utilizing electrophysiology to record induced-membrane potentials, whole-cell currents, and single ion channel currents (Figure 1C). Previous neuronal cultures using midgastrula stage embryos exhibited electrical activity at day 3 to day 9 [28], [29] in contrast to day 1 and day 2 cultures, which did not show electrically excitable cells [31]. Furthermore, neuronal cultures from third-instar larval brains showed sensitivity to the sodium channel neurotoxin, Veratridine at day 4, suggesting that by day 4 these neurons reached electrical maturity [22]. Therefore, we also performed electrophysiological analysis of our neuronal cultures at day 4 to evaluate the functionality of our cultures and to demonstrate their ability to retain their normal properties as they grew over time.

We used Ba^2+^ ions to perform current-clamp recordings [22] and as illustrated in Figure 1CI upon injection of positive current pulses beyond 100 nA, an action potential-like rapid membrane depolarization could be elicited. We attribute this behavior to the active membrane properties of the neuron. Voltage-clamp analysis was used to investigate the K^+^ conductance in our cultured neurons. Upon application of step depolarizing pulses, a delayed outwardly-rectifying K^+^ current (Figure 1CII) was observed. The presence of functional neuronal nicotinic acetylcholine receptors (nAChR) was also observed using the single channel patch clamp in the ‘on cell’ configuration in response to 100 µM ACh. A representative current recording of the AChRs is shown in Figure 1CIII. The conductance of the channels was ∼70 pS (3 patches). Together these results suggest that our primary neurons are functional and have the ability to generate active non-canonical action potentials. Moreover, we rationalize that since ion channels are transported by kinesin-1 motors [32] (Figure S3BC), the eliciting of electrophysiological responses would suggest that the vesicle transport pathway is operating properly in these neurites.

We further assessed the localization of synaptic markers, since previous work done in mammalian cultures has shown that presynaptic (synaptophysin) and postsynaptic markers (GABA_A_ β2/3 subunit) colocalized in clusters at growth cones indicating the formation of short-lived aggregates of pre- and postsynaptic proteins during early synapse formation [35]. Consistent with this, we also observed discrete colocalization of an antibody for horseradish peroxidase (HRP) which binds to neuronal membranes in *Drosophila* and serve as a neuronal marker [36], [37], [44] that localizes in presynaptic terminals and axons [38], with postsynaptic density protein-95 homologue discs large (DLG) [39] at neurite growth cones (Figure S3A). DLG is a scaffolding protein involved in synaptic anchoring to modulate postsynaptic membranes during synapse formation [40]. Therefore, the clustering of these neuronal proteins is consistent with observations of presynaptic and postsynaptic protein clustering that occurs prior to synapse formation in early synaptogenesis [35]. Additionally, bruchpilot (BRP) a presynaptic protein essential for establishing active zones [41] and glutamate receptor subunit GluN2A, which is usually seen in the post synapse [42] also showed colocalization with HRP (Figure S3BC) at neurite growth cones. Together, these results indicate that while these neuritis contained growth cones at their terminals, neurons in culture can express the necessary proteins to potentially form functional synapses at later time points.

We further characterized our neuronal cultures by using immunofluorescence and several neuronal antibodies. Cell body nuclei were observable with DAPI, while HRP, which binds to neuronal membranes [36], [38], [43], [44] visualized neuronal projections [45]. Futsch, the MAP1B protein [23], [24], [46], was observed to be enriched in axonal neurites and growth cones relative to the rest of neuron (Figure S2A). Similarly, only segmental nerves and neuromuscular junctions (NMJ) showed prominent staining in whole mount larvae (Figure S5). SUK4 against kinesin heavy chain showed localization in the entire neuron with the cell body containing the highest levels, similar to what was seen in larvae (Figure S4A, S5ABC). Cystein String Protein (CSP), a critical SNARE component involved in vesicle recycling [46]–[48] was found in the cell bodies, and projections (Figure S4B). In larvae, CSP was also found in the segmental nerves, NMJ and ventral ganglion (Figure S5ABC) [49]. Syntaxin, an integral component in the SNARE complex promoting vesicle-to-plasma membrane fusion [50] was found in neuritis and in growth cones (Figure S4C). Similarly, syntaxin was found in segmental nerves and NMJ (Figure S5ABC). Discs large, a synaptic protein anchoring and trafficking [39], [40], [51], [52], was found with relatively uniform expression throughout the neuron (Figure S4D) similar to larval neurons (Figure S5ABC). Phosphorylated cJun N-terminal Kinase (p-JNK) localization was found throughout the neuron (Figure S4E), complementary to larval nerves (Figure S5ABC) [53]. An antibody against Choline Acetyltransferase (ChAT), a cholinergic neuron marker, was localized in high concentrations to the cell body and minimally in projections and growth cones (Figure S4F), similar to what was previously observed in whole mount larvae [54]–[56] (Figure S5ABC). Highwire, a negative regulator of NMJ growth [57]–[59], localized to neuronal cell bodies and growth cones, and minimal localization was seen within projections (Figure S4G). Similarly, in whole mount larvae, Highwire was prominently observed in the ventral ganglion and the NMJs, while faint staining was observed in the segmental nerves (Figure S5ABC) [46], [60]. Taken together, our immunolocalization analysis indicated that our primary neurons express a variety of synaptic proteins similar to larval segmental nerves, and retain their physiological properties.

### Spatial and Temporal Characteristics of Vesicle/Organelle Transport in *Drosophila* Primary Neurons

The general movement dynamics of GFP tagged vesicles have been described in whole mount *Drosophila* larvae, but how normal transport dynamics or transport defects contribute to the growth of neurons and their function is unclear due to the fact that the whole-mount larval preparation only evaluates the spatial characteristics of vesicle dynamics [9]–[11], [61], [62]. While previous studies have attempted to assess the temporal aspects of mitochondrial transport in vertebrate neurites (chicken DRG neurons [63], rat cortical neurons [64], mouse sciatic nerve [65]), these studies did not fully assess the intricate transport behaviors providing an incomplete view of temporal changes in mitochondrial transport. Therefore, we systematically evaluated both the spatial and temporal characteristics of six vesicles/organelles using our primary neuronal cultures and a custom particle tracking program [9], [10].

Vesicle transport analysis in mammalian cultures has typically been done at day 2 [33], [34], when neurites have not yet established functional synapses. To this end we also used day 1 and day 2 axonal neurites for motility analysis. To systematically characterize the spatial and temporal aspects of vesicle/organelle motility and how these contribute to neuronal growth, we generated primary neuronal cultures from larval brains expressing five vesicle proteins, human amyloid precursor protein (APP-YFP), atrial natriuretic factor (ANF-GFP), synaptotagmin (SYNT-EGFP), synaptobrevin (SYNB-GFP) and human transferrin receptor (HTFR-GFP), and one organelle protein mitochondria, (MITO-GFP), using the APPL-GAL4 driver. In Drosophila larvae, APPL is enriched in three thoracic neuromeres (t1, t2, t3) and in the eighth abdominal neuromere (a8) [66], and therefore GFP-expressing neurons comprised roughly half of the neurons in our primary culture (47.4%–66.1% at day 2, Table S2). Similar to transport analysis in mammalian neuronal cultures [33], [34], [67], [68], imaging for quantitative analysis of transport dynamics was done from single isolated neurons at day 2, since after day 2 neuronal projections grew to such lengths resulting in the overlap of neurons and intersection with other neurons.

We first evaluated whether expression of various GFP-tagged proteins had an effect on neuronal growth rates, by measuring the cell body diameter and the longest neuronal projection length over 2 days. Although more than 10 neuronal cells were imaged, measurements were taken from 10 randomly selected neurons for each genotype at day 1 and day 2 from 5 independently generated cultures. While neurons expressing various GFP-tagged proteins grew similar to neurons not expressing GFP proteins (Figure S6AB, Table S1), we did not find a significant difference in neurite lengths or cell body diameters between the different neurons expressing various GFP tagged proteins or between GFP expressing neurites and non-expressing neurites. However, some significant changes were observed in the number of neuronal branches between the different neurons expressing the GFP tagged proteins compared to non-expressing neurites (Figure S6C, Table S3). While at day 2 all neurons expressing GFP tagged proteins grew (Figure S6AB, Table S1), significant decreases in the number of branches in neurons expressing APP, ANF, SYNT, HTFR and MITO were seen compared to non-expressing neurites (Figure S6C, Table S3). Interestingly, only SYNB-GFP neurons showed a trend towards increased branching at day 2 (Figure S6C, Table S3).

**Figure 2:**
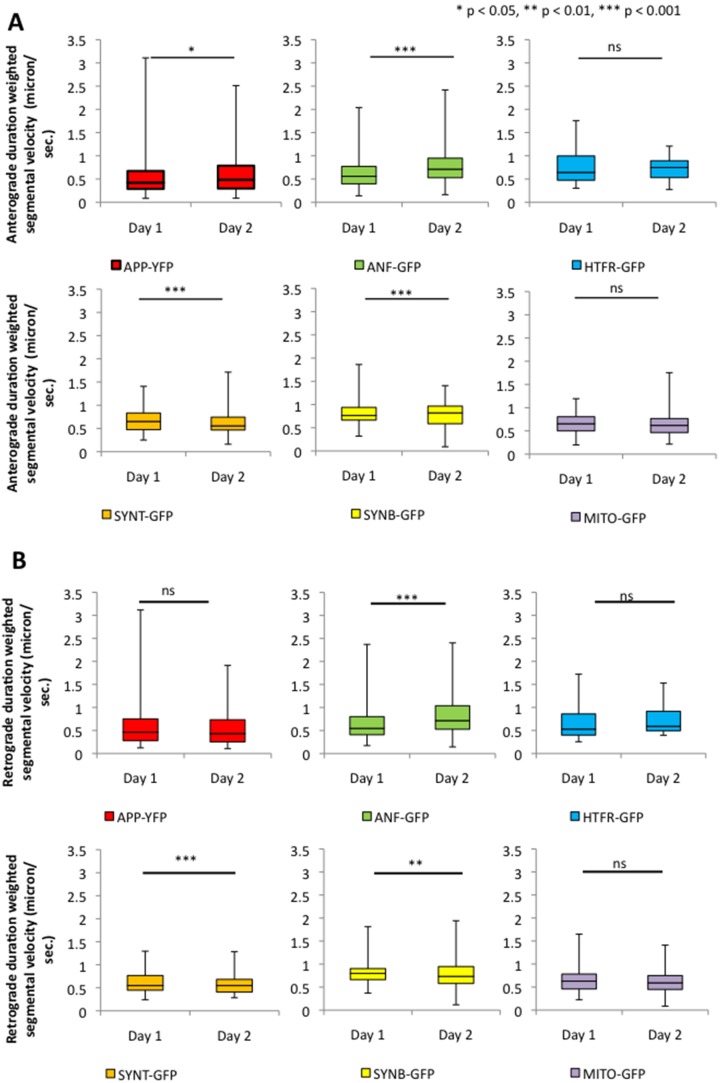
Anterograde and retrograde velocities of GFP/YFP tagged vesicles/cargo significantly change temporally. The average duration weighted segmental velocities of six vesicles/organelle revealed significant shifts in their movement dynamics at day 2 compared to day 1. Box-plots outline the distribution of vesicle velocities. Upper and lower box edges represent 75^th^ percentile and 25^th^ percentile respectively. Upper and lower whiskers represent the maximum and minimum of vesicle velocities respectively. The horizontal line represents the median of vesicle velocities. (**A**) The anterograde duration-weighted segmental velocity of APP-YFP vesicles significantly increased at day 2 (p = 0.015). Anterograde segmental velocity distributions of ANF-GFP vesicles also significantly increased at day 2 (p = 1.068E-6). Strikingly, anterograde segmental velocity of SYNB-GFP vesicles and SYNT-GFP vesicles significantly decreased over time (p = 3.484E-6 and p = 0.0004, respectively). HTFR-GFP and MITO showed no significant change at day 2 compared to day 1. (**B**) The retrograde duration-weighted segmental velocity of ANF-GFP significantly increased (p = 1.092E-7), while SYNB-GFP significantly decreased over time (p = 6.043E-7). APP-YFP, HTFR-GFP, SYNT-GFP, and MITO-GFP showed no significant changes at day 2 compared to day 1. N = 10 cells; *p<0.05, **p<0.01, ***p<0.001 by Wilcoxon-Mann-Whitney rank sum test for nonparametric distributions. NS = not significant.

To evaluate the spatial and temporal motility dynamics of the six GFP tagged vesicles/organelle, we imaged vesicle motility at day 1 and 2 and analyzed motility in the longest neurite. To quantify vesicle motility we utilized our customized particle tracking software program to analyze data collected at a spatial resolution of 0.126 microns/pixel and a temporal resolution of 0.2 seconds per frame [9]–[11]. All GFP tagged vesicles/organelle showed robust bi-directional movement (Figure 2AB, S7). At day 1, APP-YFP vesicles moved at an average duration-weighted segmental velocity of 0.331±0.083 micron/sec and 0.355±0.077 micron/sec in the anterograde and retrograde directions respectively, while at day 2 APP-YFP vesicles moved at an average velocity of 0.386±0.091 microns/sec and 0.355±0.079 microns/sec in the anterograde and retrograde directions respectively (Figure 2AB). Only the anterograde vesicles showed a significant increase between day 1 and day 2 (p_antero_ = 0.015, p_retro_ = 0.997; Figure 2AB; Table S4). The duration-weighted segmental velocity reports the average velocity of vesicle segments during their entire time of movement (see Methods). A segment is defined as the portion of a vesicle trajectory within the sample period in which there is no pause or reversal. Thus, the velocities of longer segments are given higher weights [9]–[11]. The unweighted segmental velocities showed similar trends (Figure S9AB). At day 2, no significant changes to the population of APP-vesicles were seen compared to day 1 (Figure S8A; Movie S3).

Interestingly, in ANF-GFP expressing cultures, significant increases in both anterograde and retrograde ANF vesicle velocities were seen. At day 1, ANF-GFP vesicles moved at an average duration-weighted segmental velocity of 0.768±0.073 microns/sec and 0.770±0.082 microns/sec in the anterograde and retrograde direction respectively, while at day 2 ANF-GFP moved at an average velocity of 0.952±0.093 microns/sec and 0.980±0.094 microns/sec in the anterograde and retrograde directions respectively (p_antero_ = 1.068E-6, p_retro_ = 1.092E-7; Figure 2AB, S7; Table S4). Similar changes were observed for unweighted segmental velocity distributions (Figure S9AB). At day 2 significant increases in the population of retrograde ANF vesicles were seen with decreases in reversing vesicles (Figure S8B; Movie S4).

At day 2 significant decreases in anterograde SYNT-vesicle velocities were observed. At day 1, SYNT-GFP vesicles moved at an average duration-weighted segmental velocity of 0.875±0.069 microns/sec and 0.768±0.063 microns/sec in the anterograde and retrograde directions respectively, while at day 2 SYNT-GFP vesicles moved at an average velocity of 0.720±0.051 microns/sec and 0.700±0.111 microns/sec in the anterograde and retrograde directions respectively (p_antero_ = 0.0004, p_retro_ = 0.055; Figure 2AB, S7; Table S4; Movie S7). The same temporal change was observed in unweighted segmental velocity populations (Figure S9AB). Interestingly, only the anterograde SYNT-vesicle population significantly decreased by day 2 while the population of other SYNT-vesicles (retrograde, reversing, or stalled) did not change (Figure S8D).

Significant decreases in both anterograde and retrograde SYNB-vesicles were observed at day 2. At day 1 SYNB-GFP vesicles moved at an average duration-weighted segmental velocity of 0.591±0.089 microns/sec and 0.507±0.074 microns/sec in the anterograde and retrograde directions respectively while at day 2 SYNB-GFP vesicles moved at an average velocity of 0.251±0.071 microns/sec and 0.265±0.035 microns/sec in the anterograde and retrograde directions respectively (p_antero_ = 3.48E-6, p_retro_ = 6.043E-7; Figure 2AB, S7; Table S4). The same changes were observed in unweighted segmental velocity populations (Figure S9AB). No significant changes in vesicle populations were observed for SYNB-GFP vesicles (Figure S8C; Movie S6).

No significant changes in HTFR vesicle velocities were observed at day 2. At day 1 HTFR-GFP vesicles moved at an average duration-weighted segmental velocity of 0.422±0.076 microns/sec and 0.427±0.096 microns/sec in the anterograde and retrograde directions respectively, while at day 2 HTFR-GFP vesicles moved at an average velocity of 0.472±0.059 microns/sec and 0.465±0.072 microns/sec in the anterograde and retrograde directions respectively (p_antero_ = 0.2651, p_retro_ = 0.409; Figure 2AB, S7; Table S4). This result was recapitulated in the unweighted segmental velocity analysis (Figure S9AB). Interestingly, a significant decrease in the anterograde HTFR vesicle population was seen at day 2 with no significant changes in the retrograde, reversing, or stalled populations (Figure S8E; Movie S5).

Surprisingly, no significant change in the anterograde and retrograde MITO-GFP velocities were observed between day 1 and day 2 (p_antero_ = 0.211, p_retro_ = 0.269; Figure 2AB, S7; Table S4). At day 1 MITO-GFP moved at an average duration weighted segmental velocity of 0.425±0.059 microns/sec and 0.420±0.063 microns/sec in the anterograde and retrograde directions respectively, while at day 2 MITO-GFP moved at an average velocity of 0.388±0.060 microns/sec and 0.389±0.063 microns/sec in the anterograde and retrograde directions respectively. This was also observed in the unweighted segmental velocity analysis (Figure S9AB). Interestingly, MITO-GFP populations also did not change temporally (Figure S8F; Movie S8). Perhaps these results may suggest that more than adequate amounts of mitochondria are transported along axons for the maintenance and function of axons as well as for the establishment and maintenance of synapses, which is in agreement with a recent study that showed that the energy requirement for vesicle transport is independent of mitochondrial ATP production [69]. Further, the temporal motility changes we observe for these vesicles/organelle may have resulted due to functional activities of the growing neuron. Therefore, taken together, our analysis reveals novel insight into the spatial and temporal characteristics of vesicle transport within a growing neuron.

While comparisons in temporal motility in larval nerves are unclear, as a step towards comparing the spatial motility behaviors *in vivo*, within larval segmental nerves in a whole organism to *in vitro* in isolated neurons, we also examined movement behaviors in larval segmental nerves expressing 5 vesicles/organelle (except synaptotagmin-GFP; Table S5). While the values of the transport parameters from our *in vivo* studies are similar to what is observed *in vitro,* we hypothesize that the observed differences in velocities seen *in vivo* vs *in vitro* may be due to physiological regulatory mechanisms present within the intact animal that are absent in an isolated neuron setting.

### Reduction of Kinesin-1 and Dynein Perturbs Both the Spatial and Temporal Aspects of Vesicle Transport

**Figure 3:**
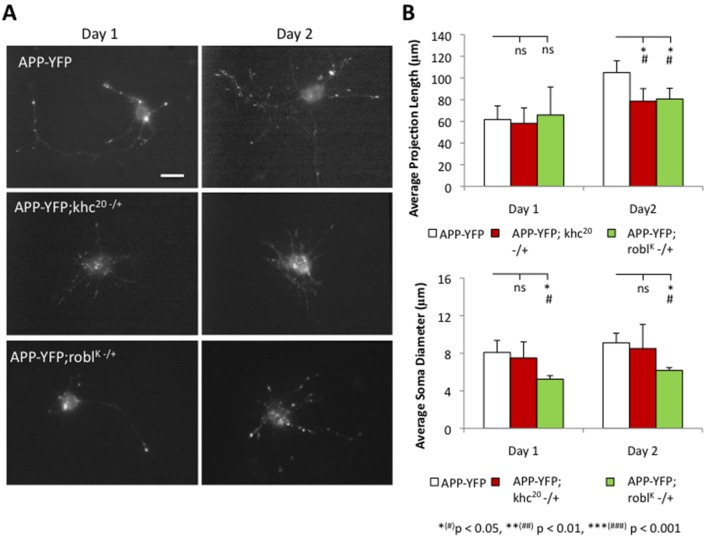
50% reduction of kinesin-1 or dynein is sufficient to perturb neuronal growth. (**A**) Representative neuronal cultures expressing APP-YFP and APP-YFP with 50% genetic reduction of either kinesin-1 or dynein are shown at day 1 and day 2. Note that short neurites are observed in cultures from 50% reduction of kinesin-1 and dynein in contrast to WT cultures. Bar = 10 microns. (**B**) Quantitative analysis of neuronal growth as assayed by projection length shows significant decreases in growth with 50% genetic reduction of kinesin-1 and dynein. At day 1 no significant difference in neurite length was observed between neurites containing 100% motor proteins, 50% kinesin-1 or 50% dynein. By day 2, neurites containing 100% motor proteins significantly outgrew neurites containing 50% kinesin-1 (p = 0.027) or dynein (p = 0.021). No significant changes were seen in the cell body diameters in neurons containing 100% motor proteins or 50% kinesin-1, but 50% reduction of dynein significantly inhibited the growth of cell bodies (p_day1_ = 0.031 and p_day2_ = 0.036). N = 10 cells; *(#) p<0.05, **(##) p<0.01, ***(###) p<0.001 by two-tailed Student t-test (*) and by Bonferroni’s test (#). NS = not significant.

In larval segmental nerves reduction of kinesin-1 and dynein motors impairs transport by decreasing vesicle motility [10], but the temporal transport defects reduction of motor proteins cause are unknown. To evaluate how reduction of motor proteins influences the temporal characteristics of vesicle transport we generated primary neuronal cultures from larvae carrying mutations of kinein-1 or dynein. In this context, the amorphic allele khc^20^ [70] or robl^K^ [71] was used in the context of APP-YFP expression. We found that heterozygous (50%) reduction of kinesin-1 or dynein was sufficient to affect neuronal growth (Figure 3AB; Table S6). Significant decreases in neurite projection length were observed at day 2 (Figure 3B; Table S6) with 50% reduction of kinesin-1 or dynein compared to 100% kinesin-1 or dynein. By day 2 50% reduction of dynein also perturbed the growth of cell bodies compared to 100% dynein. In wild type control neurons (containing 100% kinesin-1 or dynein), the average rate of axon growth was 1.80 µm/h, while 50% reduction of kinesin-1 decreased the axon growth rate to 0.85 µm/h. 50% reduction of dynein decreased the axon growth rate to 0.62 µm/h. Shorter axon lengths were only observed at day 2 for both 50% reduction of kinesin-1 or dynein (Table S6; kinesin-1 p = 0.531 and dynein p = 0.823 at day 1, kinesin-1 p = 0.021 and dynein p = 0.015 at day 2). However, 50% reduction of kinesin-1 had no significant effect on the growth of cell body diameters (p = 0.71 at day 1, p = 0.88 at day 2; Figure 3B) or cell body growth rates (Table S6), while 50% reduction of dynein significantly inhibited growth of cell bodies compared to wild type neurons (Figure 3B; Table S6; p = 0.034 at day 1, p = 0.03 at day 2). Taken together, these results suggest that reduction of motor proteins impairs neuronal growth.

**Figure 4:**
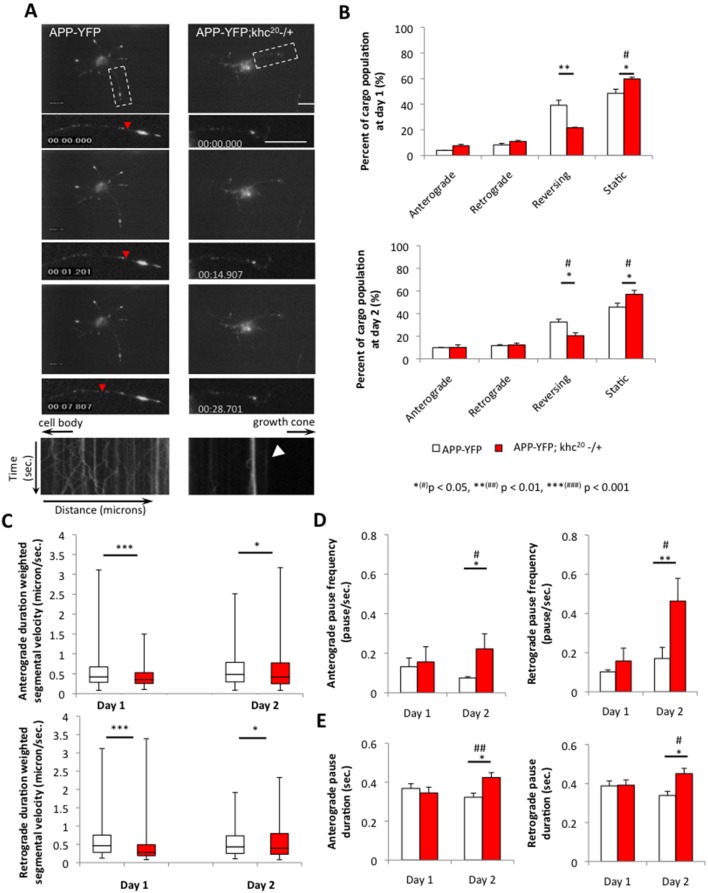
50% reduction of kinesin-1 perturbs the movement of APP vesicles. (**A**) A representative movie montage from a single neuron is shown from a primary neuronal culture expressing APP-YFP in the context of 100% kinesin-1 (APP-YFP) or 50% kinesin-1 (APP-YFP;khc^20^−/+). The axonal neurite is boxed and is enlarged with its kymograph. Note that 50% reduction of kinesin-1 (APP-YFP;khc^20^−/+) causes many stalled APP-YFP vesicles compared to neurites containing 100% kinesin-1 (arrowhead). Robust movement is seen in the kymograph with 100% kinesin-1 (APP-YFP, red arrowhead) in contrast to stalled vesicles seen in the kymograph with 50% reduction of kinesin-1. Y-axis depicts time in seconds and X-axis depicts distance traveled in microns. The cell body and growth cone directions are depicted by the arrows. Bar = 10 microns. (**B**) Quantitative analysis of the percent of cargo population indicates that 50% reduction of kinesin-1 significantly increases the percentage of stationary vesicles (p_day1_ = 0.002 and p_day2_ = 0.031) and decreases the percentage of reversing vesicles compared to 100% kinesin-1 at both day 1 and day 2 (p_day1_ = 0.013p_day2_ = 0.021) both at day 1 and day 2. (**C**) Both the anterograde (p_day1_ = 0.0002 and p_day2_ = 0.045) and retrograde (p_day1_ = 5.523E-5 and p_day2_ = 0.044) median duration weighted segmental APP velocities significantly decreased with 50% reduction of kinesin-1 compared to 100% kinesin-1 at day 1 and day 2. Upper and lower box edges represent 75^th^ percentile and 25^th^ percentile, respectively. Upper and lower whiskers represent the maximum and minimum of the data, respectively. The horizontal line represents the median. Significance was determined by Wilcoxon-Mann-Whitney rank sum test for nonparametric distributions. At day 2, both the anterograde and retrograde (**D**) pause frequencies (p_antero_ = 0.034 and p_retro_ = 0.002) and (**E**) pause durations (p_antero_ = 0.028 and p_retro_ = 0.039) significantly increased in axonal neurites containing 50% reduction of kinesin-1 compared to axonal neurites containing 100% kinesin-1. N = 10 cells; *(#) p<0.05, **(##) p<0.01, ***(###) p<0.001 by two-tailed Student t-test (*) and by Bonferroni’s test (#).Bar = 10 microns.

We next examined the motility of APP-YFP vesicles in the context of 50% reduction of kinesin-1 (khc^20^) [70], [72] or dynein (robl^K^) [71] in the longest axonal neurite. In wild type neurons containing 100% kinesin-1, no significant difference in APP-vesicle motility was seen between day 1 and day 2 (Figure 4A, Table S7). The average duration weighted segmental velocities in WT neurons were 0.336±0.087 microns/sec and 0.355±0.077 microns/sec in the anterograde and retrograde directions respectively at day 1 and 0.386±0.091 microns/sec and 0.355±0.080 microns/sec in the anterograde and retrograde directions respectively at day 2. In contrast, the motility of APP-vesicles was greatly perturbed in neurons with 50% reduction of kinesin-1 (Figure 4A) and quantification analysis revealed significant decreases in both anterograde and retrograde velocities for APP vesicles (Figure 4C; Movie S9). At day 1, the average duration weighted segmental velocities were significantly decreased from 0.336±0.087 microns/sec and 0.355±0.077 microns/sec in WT to 0.257±0.054 microns/sec and 0.273±0.059 microns/sec in 50% reduction of kinesin-1 in the anterograde and retrograde directions respectively (p_antero_ = 0.0002, p_retro_ = 5.523E-5,Table S7). At day 2 the average velocities were decreased from 0.386±0.091 microns/sec and 0.355±0.080 microns/sec in WT to 0.345±0.077 and 0.345±0.073 microns/sec in 50% reduction of kinesin-1 in the anterograde and retrograde directions respectively (p_antero_ = 0.045, p_retro_ = 0.044, Table S7). Decreases in vesicle velocities appear to be dependent on increases in vesicle pause frequencies and durations in both the anterograde and retrograde directions (Figure 4DE, Table S7). Cargo population analysis also showed an increase in the number of stalled vesicles in neurons with 50% reduction of kinesin-1 compared to WT neurons at both day 1 and day 2 (Figure 4B, Table S7). Decreases in the reversing population of vesicles were also seen in neurons with 50% reduction of kinesin-1 compared to WT neurons in both day 1 and day 2 (Figure 4B, Table S7). While these results are consistent with previous spatial observations in whole mount *Drosophila* larval preparations [10], the comparison of APP-vesicle motility between day 1 and day 2 indicates that reduction of kinesin-1 also affects vesicle motility temporally. Therefore, both the spatial and temporal defects we observe may cause the neuronal growth defects observed in kinesin-1 reduced cultures.

**Figure 5:**
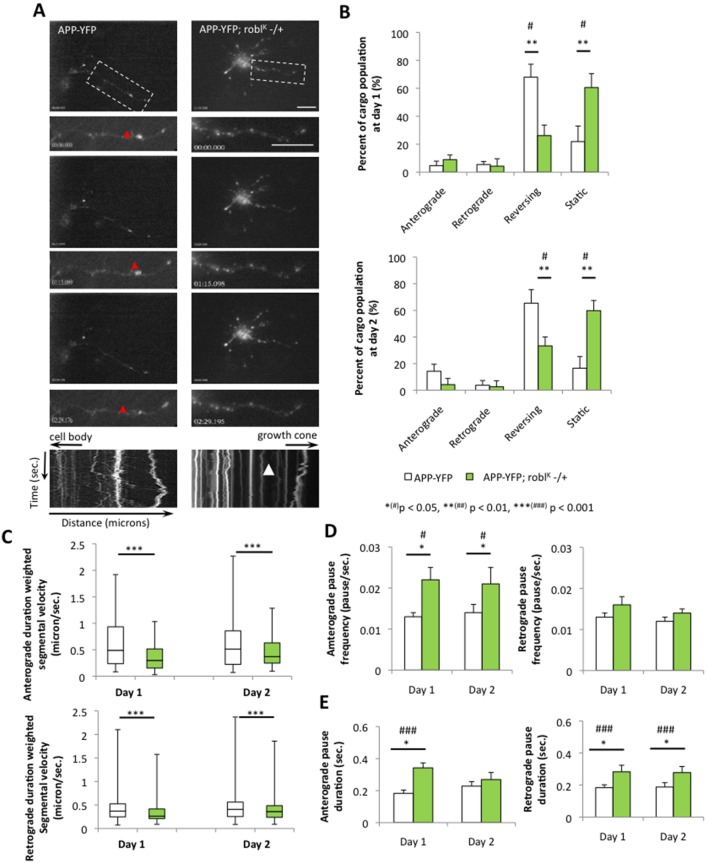
50% Reduction of dynein perturbs the movement of APP vesicles. (**A**) A representative movie montages from a single neuron is shown from a primary neuronal culture expressing APP-YFP in the context of 100% dynein (APP-YFP) or 50% dynein (APP-YFP; robl^K^ −/+). The boxed region shows the enlarged axonal neurite with its kymograph. In the kymographs, the X-axis represents the distance, while the Y-axis represents the time. The cell body and growth cone are indicated with the arrows. 50% reduction of dynein (APP-YFP; robl^K^ −/+) led to many stalled vesicles (arrowhead) in contrast to many moving vesicles observed with 100% dynein (APP-YFP, red arrowhead). (**B**) 50% reduction of dynein significantly increased the percentage of stationary vesicles (p_day1_ = 0.003 and p_day2_ = 0.002) and decreased the percentage of reversing vesicles (p_day1_ = 0.004 and p_day2_ = 0.002) at both day 1 and day 2. (**C**) Both the anterograde (p_day1_ = 1.814E-6 and p_day2_ = 0.0004) and retrograde (p_day1_ = 0.0005 and p_day2_ = 6.743E-5) APP vesicle velocities were significantly decreased with 50% reduction of dynein compared to 100% dynein at both day 1 and day 2. Upper and lower box edges represent 75^th^ percentile and 25^th^ percentile, respectively. Upper and lower whiskers represent the maximum and minimum of the data, respectively. The horizontal line represents the median. Significance was determined by Wilcoxon-Mann-Whitney rank sum test for nonparametric distributions. (**D**) The anterograde pause frequency significantly increased at day 1 (p = 0.021) and day 2 (p = 0.042). (**E**) The anterograde pause duration significantly increased at day 1 (p = 4.284E-5) while the retrograde pause duration (p = 0.015) significantly increased at day 1 (p = 0.015) and day 2 (p = 0.043) compared to axonal neurites containing 100% dynein. (N = 10 cells; *(#) p<0.05, **(##) p<0.01, ***(###) p<0.001 by two-tailed Student t-test (*) and by Bonferroni’s test (#)). Bar = 10 microns.

Similar to reductions in kinesin-1, 50% reduction of dynein also perturbed vesicle transport and decreased both anterograde and retrograde velocities of APP-vesicles in the longest neurite (Figure 5; Movie S10). Since reduction of dynein significantly affected the growth of neurons and only faint motile APP-YFP vesicles were observed (Figure 3), we imaged these cultures at a temporal resolution of 1.0 seconds per frame. For comparison, APP vesicle motility in wild type neurons was also imaged at 1.0 second per frame. At day 1 and day 2 APP-motility was significantly reduced in both the anterograde and retrograde directions in neurons with 50% dynein compared to 100% dynein (Figure 5C). Pause frequencies and durations were also increased (Figure 5DE). At day 1, the average velocities were significantly decreased from 0.060±0.014 microns/sec and 0.059±0.051 microns/sec to 0.044±0.011 microns/sec and 0.049±0.011 microns/sec in the anterograde and retrograde directions respectively in neurons with 50% reduction of dynein compared to wild type (p_antero_ = 1.814E-6, p_retro_ = 0.0005, Table S7). At day 2 the average velocities were decreased from 0.065±0.017 microns/sec and 0.074±0.021 microns/sec to 0.053±0.010 and 0.054±0.011 microns/sec in anterograde and retrograde respectively in neurons with 50% reduction of dynein compared to neurons having 100% dynein (p_antero_ = 0.0004, p_retro_ = 6.742E-5, Table S7). Cargo population analysis also showed a significant increase in the number of stalled vesicles in neurons with 50% reduction of dynein compared to WT neurons at both day 1 and day 2 (Figure 5B, Table S7). Significant decreases in the reversing population of vesicles were also observed in neurons from 50% reduction of dynein compared to WT neurons in both day 1 and day 2 (Figure 5B, Table S7). Taken together, our analysis indicates that reduction of motor proteins perturbs vesicle transport both spatially and temporally, and these attributes likely contribute to the defects seen in neuronal growth. Thus, our systematic analysis using six vesicles/organelle demonstrates the temporal aspects of vesicle transport and provides insight into its long-term effects.

### Some Vesicle Blockages are Dynamic and Resolve Over Time, While Others are Static

Third instar larvae carrying loss of function mutations of kinesin-1 or dynein show a characteristic neuromuscular phenotype and are larval lethal [4], [71]–[74]. These mutant larvae flip their tail upward indicating early paralysis of posterior-ventral body wall muscles. Previous analysis showed that the segmental nerves from these animals contained prominent accumulations of the synaptic vesicle protein, cystein string protein (CSP) [4], [71]–[74]. These accumulations are referred to as vesicle blockages. Under transmission EM many types of identifiable axonal cargo, namely mitochondria, clear vesicles, dense core vesicles, large multi-vesicular bodies, and large, dark polysomal vacuoles were contained within these massive axonal swellings [4], [49]. Since these blockages were identified in fixed larvae, little is known about the spatial and temporal characteristics of these blockages.

**Figure 6:**
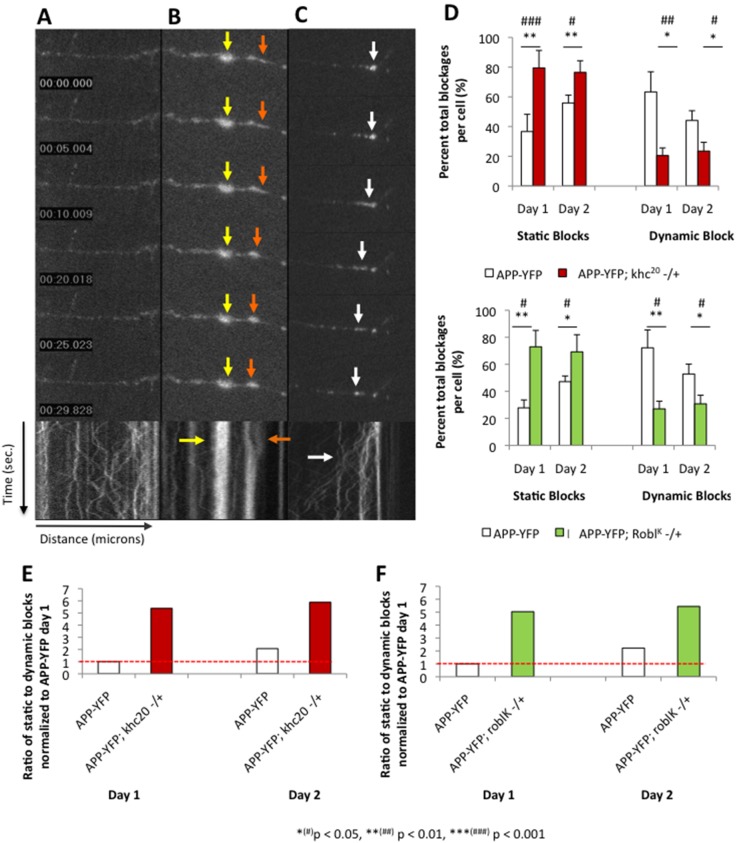
Two types of vesicle blockages are observed, static non-moving blocks that do not resolve over time and dynamic, clusters of vesicles that resolved over time. (**A**) In control neurites robust APP-YFP motility is observed with no vesicle blockages. A representative movie montage is shown with its kymograph. X axis = time (seconds), Y axis =  distance (microns). (**B**) A representative axonal neurite shows a static non-moving APP block (yellow arrows) that do not resolve temporally (arrow in kymograph). In the same axonal neurite a static block being formed can be observed (orange arrows). (**C**) A representative axonal neurite shows a dynamic block that resolves over time (white arrow). Note that in the kymograph the block can be observed resolving over time and single vesicle tracks are seen over time (white arrow). Bar = 10 microns (**D**) Quantitative analysis of the percent total blockages per cell is depicted from day 1 to day 2 for static and dynamic blocks. Note that 50% reduction of kinesin-1 or dynein significantly increases the percent of static, non-moving blocks (kinesin-1 p_day1_ = 0.015; dynein p_day1_ = 0.010 and p_day2_ = 0.022), while the number of dynamic, motile blocks decreases (kinesin-1 p_day1_ = 0.005 p_day2_ = 0.033; dynein p_day1_ = 0.009 and p_day2_ = 0.022). (**E**) Normalized ratio of static to dynamic blocks shows that 50% reduction of kinesin-1 causes more static blocks compared to dynamic blocks in contrast to axonal neurites containing 100% kinesin-1 at both day 1 and day 2. (**F**) The normalized ratio of static to dynamic blocks indicate that 50% reduction of dynein also causes more static blocks compared to dynamic blocks at day 1 and day 2. Y-axis  =  ratio of static to dynamic blocks normalized to APP-YFP at day 1 in arbitrary units (AU), X-axis  =  genotype at day 1 and day 2. N = 10 cells; *(#) p<0.05, **(##) p<0.01, ***(###) p<0.001 by two-tailed Student t-test (*) and by Bonferroni’s test (#).

Since previous work has shown that larvae expressing APP in the context of 50% reduction of kinesin-1 and dynein contained many vesicle blockages [49], to spatially and temporally evaluate blockages, we generated APP-YFP expressing neuronal cultures in the context of 50% reduction of kinesin-1. While these cultures contained vesicle blocks, surprisingly we observed that some of the APP-YFP containing blockages were not static blocks as previously thought, but were dynamic clusters of vesicles that changed over time. At least two types of vesicle blockages were observed (Figure 6, arrows), those that were static and rarely changed over time, and those that were dynamic and resolved over time. Over time, static blockages did not move, dissolve or dissociate within our imaging window (Figure 6B, yellow arrow), while dynamic blockages showed motility along the neurite projection and were able to dissociate over time (Figure 6B, orange arrow; Figure 6C, white arrow). To further characterize the formation and maintenance of these blocks temporally, we quantified and compared the numbers of these two types of blockages in APP-YFP expressing cultures in the context of 100% motor proteins or 50% motor proteins. Strikingly, we found that over time, the number of dynamic blocks decreased, while the number of static blocks increased with 50% reduction of kinesin-1 (Figure 6D) or with 50% reduction of dynein (Figure 6D) compared to 100% kinesin-1 or dynein. This same phenomenon was also observed in larval segmental nerves *in vivo* suggesting that this observation is not an artifact or is restricted to neuronal cultures (data not shown).

Given the identification of static and dynamic blockages, we sought to gain further insight into how these blockages form and how each may contribute to cellular toxicity. In this context we hypothesized that static blockages would impede transport and, over time, would perturb vesicle motility on several microtubule tracks creating larger blockages. In contrast, we hypothesized that dynamic blockages were temporary and would dissolve over time allowing subtle changes in transport that were harmless. However, over time, prolonged subtle changes to normal transport could tip the balance causing static blockages to form enabling conditions for cellular toxicity. Furthermore, while static blockages may be more detrimental to cell survival, dynamic blockages may not be harmful at first but may have the propensity to become harmful as they change to become static blockages over time. To test predictions of this hypothesis, we first assessed the ratio of static to dynamic APP-YFP blockages in neurites expressing APP-YFP with 100% kinesin-1 or dynein and in neurites expressing 50% reductions in kinesin-1 or dynein at day 1 and day 2. We found that reduction of kinesin-1 caused a higher static to dynamic block ratio at both time points compared to neurites containing 100% kinesin-1 (Figure 6E; Table S8). In APP-YFP expressing neurons, the normalized ratio of static to dynamic blockages increased from 1.00 arbitrary units (AU) at day 1 to 2.01 AU at day 2 (Figure 6E, F; Table S8). With 50% reduction of kinesin-1, the normalized static to dynamic blockage ratio increased to 5.38 AU at day 1 compared to 1.00 AU for cultures containing 100% kinesin. At day 2 the normalized static to dynamic blockage ratio was 5.88 AU with 50% reduction of kinesin-1 compared to 2.01 AU at day 2 for cultures containing 100% kinesin. Similarly, compared to wild type neurons, neurons with 50% reduction of dynein also had higher static to dynamic block ratios at both time points (Figure 6F; Table S8). At day 1 the ratio was 5.03 AU compared to 1.00 AU for cultures containing 100% dynein. At day 2 the normalized static to dynamic blockage ratio was 5.44 AU compared to 2.01 AU at day 2 for cultures containing 100% dynein (Figure 6F; Table S8). Taken together, these observations suggest that static blocks occur more commonly in neurons containing reductions of motor proteins compared to wild type neurons. Furthermore, over time the propensity to form static blocks also increased.

To further evaluate the probability that a static blockage is likely to form at a later time, we calculated the odds ratio (OR) of static block formation over time for each genotype (Table S9). The odds ratio is a measure of the association between an exposure or risk variable with an outcome. It is used to determine whether a particular variable is a risk factor for the outcome of interest as well as to assess the magnitude of the risk factor and is frequently used to assess the occurrence of disease outcomes given exposure to a variable of interest in case-control and cross-sectional studies [75]. Here, we used the OR to compare the probability of static block formation at day 2 relative to day 1. At all time points, neurons with motor protein reductions were more likely to have static blocks than wild type neurons (Table S9). Compared to wild type, 50% reduction in kinesin-1 led to a 6.69 fold increase in the probability that a block will be static at day 1 and a 2.57 fold increase in the probability that a static block will form at day 2 (OR_day1_ = 6.69, p = 2.3E-3; OR_day2_ = 2.57, p = 0.001, Table S9). Similarly, 50% reduction in dynein led to a 7.02 fold increase in the probability that a block will be static at day 1 and a 2.52 fold increase in the probability that a static block will form at day 2 (OR_day1_ = 7.02, p = 2.3E-3; OR_day2_ = 2.25. p = 0.001). Over time, wild type neurons expressing APP-YFP were twice as likely to form static blockages than neurons with reductions in kinesin-1 (OR = 2.19, p = 0.015; Table S9) or dynein (OR = 2.32, p = 0.001; Table S9). The probability of static blocks forming over time did not change in neurons with reductions in kinesin-1 (OR = 1.09, p = 0.09) or dynein (OR = 1.08, p = 0.413). Perhaps this is because most blocks in neurons with 50% reduction of motor proteins were already static at day 1 (Figure 6D), and temporally more static blocks will be generated over time under conditions where transport is greatly stressed, such as increased expression of APP-YFP which completes for motors [74]. Although future study is needed, our work suggests a model in which subtle changes in the temporal and spatial characteristics of vesicle transport may initially lead to benign dynamic blocks that will resolve over time (Figure S10C). However, if unfavorable conditions arise which stresses the highly regulated vesicle transport pathway then there is a greater propensity that more static blocks will form causing disruption of transport, leading to downstream cellular defects (Figure S10B).

## Discussion

We have systematically characterized both the spatial and temporal characteristics of vesicle transport in a primary neuronal culture system. Our observations indicate that our primary neurons are healthy, readily grow and are functional. Our results led us to two major findings, 1) the long-term temporal changes observed in vesicle motility may reflect functional roles in the developing neuron, and 2) vesicle blockages possess inherent dynamic motility behaviors that change over time. The novel spatial and temporal properties of vesicle transport we observe provide new insights into the mechanisms underlying axonal transport and its role in disease pathology.

### Novel Temporal Trends in Vesicle Motility May Reflect Functional Roles in the Developing Neuron

It is becoming evident that transport within axons is highly regulated and that proper transport is essential for development, playing critical roles in neuronal growth, function and maintenance. Recent studies in whole-mount *Drosophila* larvae have uncovered novel regulatory aspects of vesicle motility [9]–[11], [76], but these do not provide a complete understanding of all aspects of vesicle motility since it is unclear how defects in transport directly contribute to neuronal viability and function. In this context, our study has evaluated both the spatial and temporal aspects of transport in a primary neuronal culture system by characterizing the motility of six vesicle/organelle (Figure 2, S7; Table S4), and how these aspects contribute to neuronal growth and function.

The human amyloid precursor protein (APP) is thought to be transported in a sub class of vesicles that contain synapsin 1, TrkA, GAP43, BACE and PS, by an association with kinesin-1 [74], [77]. Previous work in whole mount *Drosophila* larvae and in cultured neuronal cells showed that APP is transported bi-directionally and distinct spatial aspects of APP motility were perturbed with reductions of kinesin-1 or dynein [10], [78]. APP vesicles can associate with kinesin-1, via its C-terminal for anterograde transport [74], [77]. Other studies suggest that APP associates with kinesin-1 via scaffolding proteins with JIPs [33], [79], [80]. Regardless of whether the APP associations with kinesin-1 are direct or indirect, we observed temporal velocity changes for anterogradely moving APP vesicles. These changes could perhaps be due to an increased need for APP at the growing synapse. Indeed recent work has shown that APP has roles in synapse growth and maintenance of synaptic structure and function [81]–[85].

Similar observations were also seen for Atrial Natriuretic Factor (ANF) vesicles (Figure 2A). ANF, a peptidergic dense core marker, is thought to be moved by both kinesin-1 and kinesin-3 in *Drosophila* and mammalian neurons [86]–[88]. Since neuropeptides are transported in dense core vesicles and are essential for maturation and maintenance of cortical circuits, perhaps the velocities of ANF-dense core vesicles may temporally change due to the interplay between kinesin-1 and kinesin-3 motors as nerve terminals begin to become established. Indeed, deficits in neuropeptides caused reduced dendritic arborization and cell body size [81]. In contrast, the anterograde synaptotagmin (SYNT) vesicle velocities significantly decreased over time (Figure 2A), and correlated to a significant decrease in the percent of anterogradely moving vesicles from day 1 to day 2 (Figure S8D). Similarly, SYNB vesicle velocities also significantly decreased over time. While SYNT vesicles are thought to be moved by kinesin-3 [89], [90], previous work has shown that cell to cell contact during synapse formation caused an increase in the accumulation of SYNT at the site of contact at the nerve terminal [91]. In addition, over expression of SYNT increased the pool of synaptic vesicles that were recruited to the synapse [92]. While excess of SYNB has been shown to increase neurite growth in cultured neurons [93], loss of SYNB caused decreases in excitatory synaptic currents eliminating evoked transmitter release but not spontaneous release [94], [95]. Therefore, while our analysis was done under conditions of single isolated neurons with no overlap of cells or intersection with other neurons, the temporal SYNT and SYNB velocity changes we observe may represent the requirement for SYNT or SYNB at nerve endings to promote synapse formation when neurons intersect.

While little is known about the functional role of HTFR, it is essential for neural development [96]. In particular, HTFR has been implicated in the transport of Fe^3+^ into mitochondria [97], and is a marker for recycling endosomes [98]–[100] moved via kinesin-1 and kinesin-3 [101]–[103]. Consistent with the fact that HTFR is known to localize preferentially to dendrites [104] we do not observe any significant temporal velocity changes with HTFR in axons. Surprisingly, we also failed to observe significant temporal velocity changes of mitochondria over time. Mitochondria are known to associate with Milton and Miro to form a complex that interacts with kinesin heavy chain for fast vesicle transport [105]. Perhaps these results may indicate that more than adequate amounts of mitochondria are transported along axons to supply the energy required for neuronal growth (Figure 3; Figure S8F). Intriguingly, recent observations have shown that the energy requirement for vesicle motility was independent of mitochondrial ATP production [69]. Although future work would be needed to determine the need for ATP production in the context of temporal vesicle motility, our observations showed striking temporal changes to vesicle motility.

While our comparisons of the spatial movement behaviors of 5 vesicle/organelle in larval neurons and isolated neurons showed some changes (Table S4, S5), our observation that reduction of kinesin-1 or dynein both caused vesicle blockages and decreased the velocities of both anterograde and retrograde transport is consistent with previous work in larval neurons [9], [10]. These observations together with other studies [106]–[110] suggest that both kinesin-1 and dynein motors are present on vesicles and that their motor activities are coupled, indicating that motor behaviors follow a coordinated model [111]. Biochemical evidence has shown interactions between dynein or dynactin with kinesin-1 through dynein intermediate chain (DIC) [112]. RNAi experiments to knockdown KLC in Drosophila S2 cells demonstrated that kinesin-1 also interacts with DIC [10]. Consistent with these observations, DIC (robl^k^) reduction also impaired both anterograde and retrograde APP-YFP transport [10]. Other work suggests that dynein can function as an activator of kinesin-1 [108]–[110],[113]. Taken together, a coordination-competition model has been proposed for vesicle transport where directionality of movement by kinesin-1 or dynein is determined by the competition between kinesin-1 or dynactin binding to DIC. Thus, our observation that both anterograde and retrograde velocities were impaired upon dynein (robl^k^) reduction (Figure 4C, 5C) is consistent with this hypothesis.

### Vesicle Blockages Possess Inherent Dynamic Properties that Change Over Time

Previous work in 3^rd^ instar larvae carrying mutations of motor proteins (kinesin-1 or dynein) showed characteristic vesicle blockages in their larval segmental nerves. These blockages stained with vesicular markers and contained many types of identifiable axonal cargo, namely mitochondria, clear vesicles, dense core vesicles, large multi-vesicular bodies, and large, dark polysomal vacuoles under EM [4]. While both static and dynamic blocks have been observed in larval neurons (data not shown), how these blockages form and how these blocks affect neuronal growth are unclear. By capitalizing on our ability to continuously image neurons over time, we have now classified vesicle blockages into at least two categories: 1) static blocks, blocks that are not mobile within the imaging window and that do not resolve over time (Figure 6B), and 2) dynamic blocks, blocks that exhibit motility in the imaging window and are able to resolve over time (Figure 6C). Interestingly, under wild type conditions dynamic blocks can be observed and there is no overall effect on neuronal growth (Figure S6, Figure 6D). In contrast, when the level of kinesin-1 or dynein is reduced static blocks increase, while dynamic blocks decrease (Figure 6D) suggesting that reductions in motors perturb the transport of vesicles by causing a shift in the occurrence of static blocks relative to dynamic blocks which also contributes to defects in neuronal growth (Figure 2,4,5). Furthermore, the odds ratio indicates that dynamic blockages are more likely to become static over time, if conditions become unfavorable (Table S9). Therefore we propose that, temporally, more static blocks will be generated over time under conditions where transport is greatly stressed. While subtle changes in the temporal and spatial characteristics of vesicle transport may initially lead to benign dynamic blocks that can resolve over time (Figure S10C), if unfavorable conditions arise which affect the highly regulated vesicle transport system, then there is a greater probability that static blocks will form, causing disruption of transport leading to downstream cellular defects (Figure S10B). While further study is needed to test predictions of this model, identifying the subtle conditions that can tip the balance between the formation of dynamic and static blocks could highlight a potentially novel therapeutic pathway for early intervention, prior to neuronal loss and clinical symptoms of disease or death.

In summary, the temporal and spatial changes in vesicle transport we observed with several synaptic vesicle proteins and mitochondria indicate that these attributes are likely due to functional activities of the growing neuron. Furthermore, reductions of motors perturbed the spatial and temporal aspects of vesicle transport, and these affects likely contributed to the defects seen in neuronal growth. Thus, our study demonstrates novel spatial and temporal characteristics of vesicle transport and provides new insights into its effects on neuronal growth. Future analysis into how the spatial and temporal aspects of transport contribute to downstream pathways will provide insights into the mechanisms of how defects in transport initiate disease pathology.

## Methods

### *Drosophila* Genetics

Fly stocks were maintained on cornmeal food at room temperature. Vesicle movement was analyzed in neurons generated by crossing virgin females expressing the pan-neuronal driver APPL GAL4 with males of either UAS-EB1-YFP, UAS-APP-YFP/y, UAS-ANF-GFP, UAS-SYNT-EGFP, UAS-SYNB-GFP, UAS-MITO-GFP/cyo, or UAS-HTFR-GFP; TM3/TM6B at 29°C. Motor protein reduction were achieved by crossing either male khc^20^/cyo [70] or robl^K^/B3 [71] with virgin female APPL GAL4; B3/pin^88k^ to yield either APPL GAL4/+; khc^20^/B3 or APPL GAL4/+; robl^K^/B3, respectively. Males of these genotypes were then crossed with virgin females homozygous for UAS-APP-YFP in 29°C.

### Generating Primary Neuronal Cultures

Primary neuronal cultures were prepared as described in [20] with minor modifications. Briefly, cultures were prepared from wandering third instar larval brains. Larvae were first washed in 95% EtOH and in water to remove food/debris. Larvae were sterilized by submersion in 95% EtOH followed by washes in S10-I Medium before dissecting them in S10-I medium (87.5 mL 1X Schneider’s Insect medium, Gibco; 10 mL Fetal Bovine Serum, HyClone; 5 mg Insulin from bovine pancreas, Sigma; 0.25 mM HCl, 0.15 mM NaOH, pH = 7.0–7.2). Isolated larval brains were placed in 0.0765 mg/ml Liberase Blendzyme in Rinaldini’s saline (Roche Applied Sciences) for 1 h at 29°C to allow disaggregation and were centrifuged for 30 s at 5000 rpm. The pellet was resuspended in media and pipetted onto a substrate coated glass well-bottom petri dish (5 µl laminin, 167 µl concanavalin A, 827 µl Sigma water). Neurons were allowed to incubate for 3 h at 29°C before adding medium containing insect molting hormone 20-hydroxyecdysone (20E) at 29°C. More than 5 independent cultures containing a total of 11 genotypes (No expression, APP-YFP, ANF-GFP, SYNT-GFP, SYNB-GFP, HTRF-GFP, MITO-GFP, APP-YFP;khc−/+; APP-YFP control, APP-YFP;roblk−/+, APP-YFP control) were generated and more than 10 neurons from each of these cultures were imaged after day 1 and again at day 2. Control and motor reduction cultures were imaged blind by a lab member not involved in the preparation of cultures to minimize bias.

### Neuronal Growth Quantification

Measurements of projection length and cell body diameter were performed as described previously [21] using phase contrast images taken at 100X magnification on a Nikon T2000-E inverted fluorescence microscope. Measurements were done in Metamorph 7.0 (Meta Imaging Series) using the multi-line tool. Although more than 10 neuronal cells were imaged, 10 neuronal cells were selected for analysis and the longest projection was traced using the multi-line tool starting from its origin at the cell body to the nerve terminus. To measure the cell body diameter, a linear line was traced through the cell body end to end. For asymmetrical cell bodies, two lines were used perpendicular to each other and averaged to measure the diameter. All growth results were graphed and significance was calculated in Excel (Microsoft Corp.) using the Student’s two-tailed Student’s t-test. To account for multiple comparisons, post hoc analysis using the Bonferroni’s test was performed in SPSS Statistics 20 (IBM Corp.). To verify the sample size (N = 10 neurons) used in the study design was appropriate for determining meaningfully significant results, effect size was calculated using Cohen’s D to determine the statistical power (1-β) of each test. For all statistically significant neuronal growth p values, a sample size of 10 neurons was sufficient to reach a statistical power (α = 0.05 and β = 0.2) of 0.80 or higher. Thus, for an effect size of Cohen’s D = 1.5, based on means and standard deviations of the preliminary data, a significant difference between two means can be detected using 10 neurons per group.

### Electrophysiological Recordings

Cells were plated on 35 mm glass bottom petri dish in S10-I culture medium and grown for 4 days. Just prior to recording, culture medium was replaced with physiological saline containing 136.5 mM NaCl, 5.0 mM KCl, 2.0 mM MgCl_2_, 1.8 mM CaCl_2_, 10 mM glucose, and 10.0 mM HEPES (pH 7.1). For current-clamp recordings, 10 mM BaCl_2_ was added to saline (140 mMKCl, 2 mMMgCl_2_, 1.8 mMCaCl_2_, 10 mM HEPES). For single channel current recordings from AChR, ACh was added to the internal solution. For whole-cell K^+^ current recordings, 5 mM EGTA was added to remove Ca^2+^.

Patch pipettes were fabricated from borosilicate glass, coated with sylgard (Dow Corning) and heat polished to a resistance of 10 MΩ. Both whole-cell and single channel currents were recorded using a PC505 amplifier (Warner instruments, Hamden, CT). The current records were low-pass filtered (20 kHz for single channel and 5 kHz for whole-cell currents) and digitized at a sampling frequency of 50 kHz (20 kHz for whole-cell) using a SCB-68 data acquisition board (National instruments, Austin, TX). Whole-cell K^+^ currents were elicited by step membrane depolarization and action potentials were recorded in current-clamp mode by injecting step current pulses holding the membrane at the resting membrane potential (RMP). The RMP of the neurons were ∼−60 mV (n = 7). Single channel current was recorded in the cell-attached mode by hyperpolarizing the membrane to −70 mV. Data were analyzed by QStudio (SUNY at UB), QuB (http://www.qub.buffalo.edu) and Sigmaplot.

### Immunohistochemistry

Two day old cultures were fixed in 8% paraformaldehyde for 30 min at 4°C. After three washes in 1X PBT cultures were incubated in primary antibody against either Bruchpilot (nc38, 1∶5, Developmental Studies Hybridoma Bank), GluRN2A (1∶100, provided by Dr. DiAntonio [42]), cysteine string protein (CSP, 1∶10, Developmental Studies Hybridoma Bank), Syntaxin (8C3, 1∶5, Developmental Studies Hybridoma Bank), Highwire (6H4, 1∶5, Developmental Studies Hybridoma Bank), Futsch (22C10, 1∶5, Developmental Studies Hybridoma Bank), Discs Large (4F3, 1∶5, Developmental Studies Hybridoma Bank), Choline Acetyltransferase (ChAT4B1, 1∶5, Developmental Studies Hybridoma Bank) or kinesin heavy chain antibody (SUK4, 1∶5, Developmental Studies Hybridoma Bank). Cultures were incubated in Alexa 488 anti-mouse (1∶100, Invitrogen), HRP-TR (1∶50, Jackson Immunoresearch Laboratories), and/or DAPI (1∶20, Invitrogen) for 2 hrs at room temperature. Cultures were mounted using Vectashield mounting medium (Vector Labs) and imaged using a Nikon TE 2000-E inverted microscope at 60X and 100X.

### Larval Preparation and Immunohistochemistry

Third instar larvae were dissected, fixed, and immunostained as described [74]. Briefly, larvae were dissected in dissection buffer (2X stock contains 128 mM NaCl, 4 mM MgCl_2_, 2 mM KCl, 5 mM HEPES, and 36 mM sucrose, pH 7.2). Dissected larvae were fixed in 4% formaldehyde and incubated with primary antibodies against either cysteine string protein (CSP, 1∶10, Developmental Studies Hybridoma Bank), Syntaxin (8C3, 1∶5, Developmental Studies Hybridoma Bank), Highwire (6H4, 1∶5, Developmental Studies Hybridoma Bank), Futsch (22C10, 1∶5, Developmental Studies Hybridoma Bank), Discs Large (4F3, 1∶5, Developmental Studies Hybridoma Bank), Choline Acetyltransferase (ChAT4B1, 1∶5, Developmental Studies Hybridoma Bank) or kinesin heavy chain antibody (SUK4, 1∶5, Developmental Studies Hybridoma Bank) overnight. Larvae were incubated in HRP-TR and secondary antibody (Alexa anti-rabbit 488, 1∶100, Invitrogen) for 2 hrs at room temperature, mounted using Vectashield mounting medium (Vector Labs) and imaged using a Nikon TE-2000E inverted microscope at 60X.

### Vesicle Imaging and Analysis

Vesicle imaging in larvae and neuronal cultures was done using a Nikon TE-2000E inverted fluorescence microscope and Metamorph Imaging software. From each culture, 10 neuronal cells were randomly selected for analysis. For all genotypes the longest neurite identified during neuronal growth quantification by measuring neurite length was used for vesicle analysis. Our analysis was done under several genetic conditions (6 GFP tagged proteins, none-expressing condition, reductions of kinesin-1 and dynein and consistent control conditions, a total of 11 genotypes and controls). Furthermore each experiment was done more than 5 times (independent) and images obtained from more than 10 cells. Consistent with others in the field we use 10 cells from at least 5 independent experiments for our detailed analysis of spatial and temporal vesicle motility. Other studies in neuronal cultures have used a range of sample sizes, N = 3 to N = 10 cells to analyze vesicle motility dynamics and have analyzed a total of 30–200 vesicles [78], [114]–[119]. We used our computational single particle tracker program to analyze the motility of over 500 vesicle (this study) and over 1000 vesicles in filleted larvae [9]–[11], [120]. For motility analysis of the 6 vesicles/organelle in larvae, 5 larvae were dissected per genotype. Four consecutive 150-frame movies were recorded from each cell at 100X (90 micron field of view) at 200 msec exposure (or 1000 msec for dynein reduction and control larvae) for a total of 2 mins were recorded per neuron at a spatial resolution of 0.126 micron/pixel. Movies were then cropped and rotated in Metamorph (Meta Imaging Series) and analyzed using a MATLAB 2010b-based (Mathworks) custom single particle tracking program as done in larvae [9]–[11]. Segmental velocities were defined as the mean velocity of a trajectory uninterrupted by a pause, reversal, or movie termination event. Duration-weighted segmental velocity evaluates the average velocity behavior that vesicles exhibit per time spent moving.

### EB1 Particle Imaging and Analysis

EB1-YFP expressing neuronal cultures were imaged using a Nikon TE-2000E inverted fluorescence microscope and Metamorph Imaging software. From each culture at day 1 and day 2, 10 neuronal cells were randomly selected for analysis. For each day, the shortest and the longest neurite identified by measuring neurite length was used for EB1 analysis. Movies were cropped and rotated in Metamorph (Meta Imaging Series) and analyzed blind using a MATLAB 2010b-based (Mathworks) custom single particle tracking program [9]–[11]. Anterograde (plus end to growth cone) and retrograde (plus end to cell body) segments were quantified from 10 cells at both day 1 and day 2 and graphed as % MT populations for axonal neurite (long neurite) and dendratic neurite (short neurite).

### Statistical Analysis

Statistical significance of mean differences in percent of cargo population was calculated in EXCEL (Microsoft Corp.) using a two-tailed Student’s t-test as they tended to follow normal distributions. Duration-weighted segmental velocity distributions often followed a mixture of normal distributions or a single normal distribution. To select the appropriate statistical test, these velocity distributions were first checked for normality using the *nortest* package of R: the Lilliefors test and Anderson-Darling test. Statistical significance of normal distributions were calculated by a two-sample two-tailed Student’s t-test while the non-normal segmental velocity distributions were compared using the nonparametric Wilcoxon-Mann-Whitney rank sum test in EXCEL and SPSS Statistics 20 (IBM Corp.). All velocity distributions were found to be non-normal. Distributions of corrected pause frequency and normalized pause duration tended to follow non-normal distributions and, thus, the Wilcoxon-Mann-Whitney rank sum test was used to calculate significance. For velocity quantification, all duration-weighted segmental velocities from each segment from all 10 neurons were pooled together before statistical analysis. This allows each particle to be considered an independent sample in the analysis to account for possible unique motor configurations on each particle [9]–[11].

To account for multiple comparisons, post hoc analysis using the Bonferroni’s test was performed in SPSS Statistics 20 (IBM Corp.). To verify the sample size (N = 10 neurons) used in the study design was appropriate for determining meaningfully significant results, effect size was calculated using Cohen’s D to determine the statistical power (1-β) of each test. Thus, for an effect size of Cohen’s D = 1.5, based on means and standard deviations of the preliminary data, a significant difference between two means can be detected using 10 neurons per group.

### Vesicle Blockage Quantification and Statistical Analysis

*In vitro* APP-YFP blockages in axonal neurites expressing APP-YFP with 100% motor proteins or in the context of 50% motor proteins were quantified from time lapsed videos of 150-frames imaged at 200 msec (50% kinesin-1 reduction and control) for a total of 30 sec per movie or 1000 msec (50% dynein reduction and control) for a total of 150 sec per movie. Movies were taken at a 2×2 binning factor giving a spatial resolution of 0.126 micron/pixel. Four consecutive movies were imaged per cell. Ten randomly selected neuronal cells were imaged from each genotype. Blockages were classified as static (exhibiting no motile behaviors within the imaging window in all four consecutive movies per cell) or dynamic (forming, dissolving, or exhibiting any motile behavior in the imaging window in all four consecutive movies per cell). Blockages found in neurites that that also exhibited blebs or swellings were excluded from analysis. The average ratio of static to dynamic blocks was determined by dividing the average number of static blocks per cell by the average number of dynamic blocks per cell at a given time point for each genotype. Each value was normalized to the corresponding wild type value at day 1. To determine the odds ratio [75], the probability of static blockage formation was determined for each time point at each genotype by dividing the number of static blocks per cell by the summed total of all blockages (static and dynamic) per cell. Similarly, the probability of non-static (dynamic) blockage formation was determined for each time point at each genotype by dividing the number of dynamic blocks per cell by the summed total of all blockages (static and dynamic) per cell. Odds ratios (OR) were used to compare the probability of static block formation at day 1 and day 2.



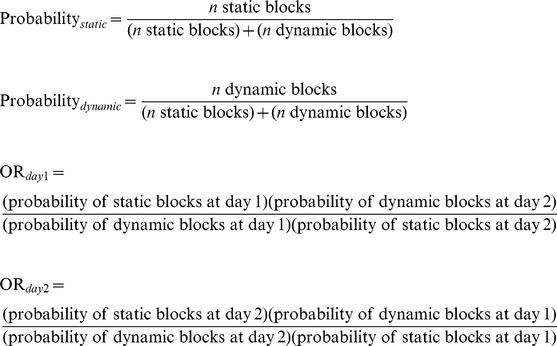



The corresponding p-values for blockage ratios were determined by binary logistical regression analysis. Static block probability was the dependent variable regressed against either static block probability in the same genotype at later timepoint or static block probability in the same timepoint in the corresponding motor reduction genotype as independent covariates. Data was analyzed in SPSS Statistics 20 (IBM Corp.).

## Supporting Information

Figure S1 **Expression of GFP-tagged proteins has no effect on the long-term growth of neurons in primary cultures.** Growth analysis of WT neurons not expressing a GFP tagged protein and neurons expressing ANF-GFP show no significant difference in neurite projection growth or cell body growth from day 1 to day 4. (**A**) No significant difference in neurite length was observed between WT and ANF-GFP expressing neurons at all four days. (**B**) No significant difference was observed between WT and ANF-GFP expressing neurons. N = 10 cells; *(#) p<0.05, **(##) p<0.01, ***(###) p<0.001 by two-tailed Student t-test (*) and by Bonferroni’s test (#). NS = not significant.
(TIF)

Figure S2 **Characterization of axonal and dendritic neurites in primary neuronal cultures.** (**A**) Futsch staining is enriched in the longest neurite (large arrow) and cell bodies (arrowhead), while faint staining is observed in short neurites (small arrow) at both day 1 and day 2. Bar = 10 microns. (**B**) At both day 1 and day 2 diffused EB1-YFP is seen throughout the neuronal cell. A representative neuronal cell is shown. The kymograph from the movie of the representative neuronal cell is shown. EB1-YFP particle tracks identified by our automated particle tracking software are shown in colors on each kymograph. Note that distinct EB1-YFP bi-directional tracks are observed in the short dendritic neurite (green box), while uni-directional tracks are observed in the longer axonal neurite (red box). Quantification analysis indicates that the short dendritic neuritis contains equal amounts of anterograde (plus end to growth cone) and retrograde tracks (plus end to cell body) (green), while the longer axonal neurite contains only anterograde tracks (plus end to growth cone) (red). Y-axis = % MT population. N = 10 neuronal cells.
(TIF)

Figure S3 **Neurons in primary culture contain proteins necessary to form functional synapses.** (**A**) Two day old cultures show colocalization of HRP and DLG (discs large), at growth cones of neurites. Boxed region enlarged to show that both these proteins appear to co-localize into clusters at the growth cone (arrow). (**B**) Two day old neurons show Bruchpilot (BRP) in cell bodies and neurites. Arrow indicates co-localized clusters. (**C**) Two day old neurons show glutamate receptor subunit, GluN2A in the cell body and neurites. Arrow indicates co-localized clusters. Bar = 10 microns.
(TIF)

Figure S4 **Primary neuronal cultures show cell bodies, axonal projections and growth cones as observed by several neuronal markers.** Neuronal cultures were stained with several neuronal antibodies. All antibodies revealed strong localization in cell bodies. HRP was used as a neuronal marker. DAPI was used to reveal nuclei. (**A**) SUK4 showed uniform expression in neurite projections. (**B**) CSP was observed in all neurite projections. (**C**) Syntaxin showed strong staining at neurtie projections and growth cones (arrow). (**D**) DLG was found uniformly in all projections. (**E**) p-JNK was seen in all projections. (**F**) ChAT was found in the cell body only and was faintly seen in neurites and growth cones. (**G**) Highwire was strongly observed in the growth cone (arrow). Bar = 10 microns.
(TIF)

Figure S5 **Localization of neuronal markers in larvae.** (**A**) In the ventral ganglion, SUK 4, CSP, Syntaxin, DLG, p-JNK, and Highwire were observed. (**B**) While all antibodies were seen in larval segmental nerves, Futsch, SUK 4, CSP, Syntaxin, p-JNK, and ChAT were strongly observed in segmental nerves. (C) While all antibodies showed localization in neuromuscular junctions (NMJs), Futsch, CSP, Syntaxin, DLG, p-JNK, and Highwire were strongly observed in this anatomical structure. Bar = 10 microns.
(TIF)

Figure S6 **Expression of six GFP-tagged vesicles/organelle has no effect on neuronal growth over time.** (**A**) Representative neuronal cells show neurite growth at day 2 relative to day 1 for all genotypes. (**B**) Quantification of cell body diameter and neurite length at day 1 and day 2 revealed increased rates of growth for each genotype, similar to neurites not expressing GFP/YFP tags. Only SYNB-GFP neurons showed a significant increase in cell body size (p = 0.041). (**C**) At day 1, APP-YFP (p = 9.03E-4) and HTFR-GFP (p = 0.002) neurons had significantly less branches than non-GFP/YFP expressing neurons. At day 2, only SYNB-GFP neurons had significantly more branches such that there was no significant difference compared to GFP/YFP non-expressing neurons. Bar = 10 microns. N = 10 cells; *(#) p<0.05, **(##) p<0.01, ***(###) p<0.001 by two-tailed Student t-test (*) and by Bonferroni’s test (#). NS = not significant.
(TIF)

Figure S7 **Robust bi-directional movement of GFP/YFP vesicles/organelle is observed in primary neurons.** Representative movie montages show distinct moving vesicles/organelle for all genotypes. The boxed region is enlarged and shows the axonal neurite region used to generate the kymographs. Note that SYNB-GFP neurites show a characteristic diffused pattern in addition to discrete moving SYNB vesicles. Moving vesicle are depicted by arrowheads. Bar = 10 microns.
(TIF)

Figure S8 **Cargo populations change over time.** Cargo population analysis indicated the anterograde (ante), retrograde (retro), reversing (rev), or stationary (static) population of vesicles for all genotypes. (**A**) APP-YFP, (**C**) SYNB-GFP and (**F**) MITO-GFP populations did not significantly change temporally. However significantly more retrograde populations were observed for (**B**) ANF-GFP at day 2 (p = 0.009) and fewer reversing vesicles were observed at day 2 (p = 0.067). Significantly fewer anterograde vesicle populations were observed for (**D**) SYNT-GFP (p = 0.028) and (**E**) HTFR-GFP (p = 0.014) at day 2. N = 10 cells; *(#) p<0.05, **(##) p<0.01, ***(###) p<0.001 by two-tailed Student t-test (*) and by Bonferroni’s test (#). NS = not significant.
(TIF)

Figure S9 **Anterograde and retrograde average segmental velocities of GFP/YFP tagged vesicles/cargo significantly change temporally.** The average duration weighted segmental velocities of six vesicles/organelle revealed significant shifts in their movement dynamics at day 2 compared to day 1. (**A**) Consistent with analysis of duration weighted segmental velocities (Figure 2), the anterograde segmental velocity of APP-YFP vesicles significantly increased at day 2 (p = 0.046). Anterograde segmental velocity distributions of ANF-GFP vesicles also significantly increased at day 2 (p = 0.004). Anterograde segmental velocity of SYNB-GFP vesicles and SYNT-GFP vesicles significantly decreased over time (p = 1.658E-8 and p = 0.025, respectively). HTFR-GFP and MITO showed no significant change at day 2 compared to day 1. (**B**) The retrograde segmental velocity of ANF-GFP significantly increased (p = 0.008), while SYNB-GFP significantly decreased over time (p = 2.031E-4). APP-YFP, HTFR-GFP, SYNT-GFP, and MITO-GFP showed no significant changes at day 2 compared to day 1. N = 10 cells; *p<0.05, **p<0.01, ***p<0.001 by Wilcoxon-Mann-Whitney rank sum test for nonparametric distributions. NS = not significant.
(TIF)

Figure S10 **Proposed model for vesicle transport dynamics.** (**A**) During development, bi-directional movement of vesicles over time (T0 to T3) is essential for the maintenance of the cell. (**B**) Aggregations of vesicles form and develop into blockages as they sequester other moving vesicles in the axon and directly impede transport. These blocks may likely cause detrimental defects to neuronal growth and function. (**C**) In normal development, transient blockages spontaneously form and resolve. These blocks do not cause a significant impairment to transport. These dynamic blocks may result due to subtle changes during transport and are likely benign.
(TIF)

Table S1 **Summary of neuronal growth in primary neuronal cultures in GFP/YFP-expressing and non-expressing neurons.**
(DOC)

Table S2 **Summary of GFP/YFP neurons in primary culture.**
(DOC)

Table S3 **Summary of neuronal branching in GFP/YFP-expressing and non-expressing neurons.**
(DOC)

Table S4 **Summary of vesicle transport measurements in primary neuronal cultures using a custom single particle tracking software program.**
(DOC)

Table S5 **Summary of vesicle/organelle motility measurements in larvae using the custom single particle tracking software program.**
(DOC)

Table S6 **Summary of neuronal growth measurements with 50% motor protein reduction in primary neuronal cultures.**
(DOC)

Table S7 **Summary of all vesicle transport measurements with 50% motor protein reduction in primary neuronal cultures.**
(DOC)

Table S8 **Summary of blockage characterization.**
(DOC)

Table S9 **Summary of temporal predictor analysis of blockage type using logistic regression analysis.**
(DOC)

Movie S1 **Primary neuron expressing EB1-YFP at day 1.** Frame rate: 0.5 sec/frame. Display rate: 6 frames/sec.
(AVI)

Movie S2 **Primary neuron expressing EB1-YFP at day 2.** Frame rate: 0.5 sec/frame. Display rate: 6 frames/sec.
(AVI)

Movie S3 **Movement dynamics of APP-YFP vesicles in a primary neuron.** Frame rate: 0.2 sec/frame. Display rate: 6 frames/sec.
(ZIP)

Movie S4 **Movement dynamics of ANF-GFP vesicles in a primary neuron.** Frame rate: 0.2 sec./frame. Display rate: 6 frames/sec.
(ZIP)

Movie S5 **Movement dynamics of HTFR-GFP vesicles in a primary neuron.** Frame rate: 0.2 sec./frame. Display rate: 6 frames/sec.
(ZIP)

Movie S6 **Movement dynamics of SYNB-GFP vesicles in a primary neuron.** Note the diffuse cytoplasmic pool of SYNB protein in addition to vesicular SYNB-GFP. Frame rate: 0.2 sec./frame. Display rate: 6 frames/sec.
(ZIP)

Movie S7 **Movement dynamics of SYNT-GFP vesicles in a primary neuron.** Frame rate: 0.2 sec./frame. Display rate: 6 frames/sec.
(ZIP)

Movie S8 **Movement dynamics of MITO-GFP in a primary neuron.** Motile MITO-GFP was observed throughout the axon. Frame rate: 0.2 sec/frame. Display rate: 6 frames/sec.
(ZIP)

Movie S9 **Movement dynamics of APP-YFP vesicles in a primary neuron containing 50% reduction of kinesin-1.** Note the inhibition of neurite outgrowth and lack of APP-YFP motility within the neuron compared to normal neurons. Also note the blocked vesicles. Frame rate: 0.2 sec./frame. Display rate: 6 frames/sec.
(ZIP)

Movie S10 **Movement dynamics of APP-YFP vesicles in a primary neuron containing 50% reduction of dynein.** Note the perturbed movement of vesicles and shortened neurites compared to normal neurons. Frame rate: 1.0 sec./frame. Display rate: 6 frames/sec.
(ZIP)
