# Supplementary figures and images for: Spatial and Temporal Characteristics of Normal and Perturbed Vesicle Transport
Source: PLoS One. 2014 May 30;9(5):e97237. doi: 10.1371/journal.pone.0097237 (PMC4039462; doi:10.1371/journal.pone.0097237)

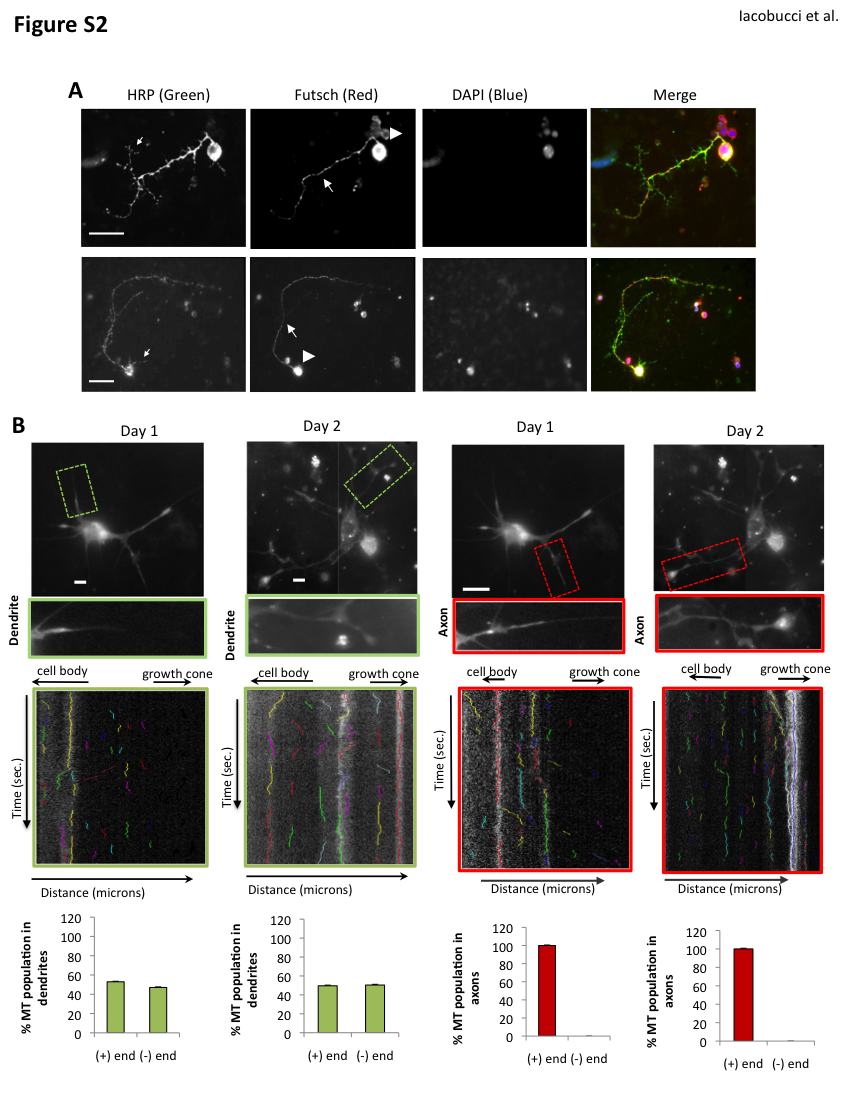

Supplement: Figure S2 — Characterization of axonal and dendritic neurites in primary neuronal cultures. (A) Futsch staining is enriched in the longest neurite (large arrow) and cell bodies (arrowhead), while faint staining is observed in short neurites (small arrow) at both day 1 and day 2. Bar = 10 microns. (B) At both day 1 and day 2 diffused EB1-YFP is seen throughout the neuronal cell. A representative neuronal cell is shown. The kymograph from the movie of the representative neuronal cell is shown. EB1-YFP particle tracks identified by our automated particle tracking software are shown in colors on each kymograph. Note that distinct EB1-YFP bi-directional tracks are observed in the short dendritic neurite (green box), while uni-directional tracks are observed in the longer axonal neurite (red box). Quantification analysis indicates that the short dendritic neuritis contains equal amounts of anterograde (plus end to growth cone) and retrograde tracks (plus end to cell body) (green), while the longer axonal neurite contains only anterograde tracks (plus end to growth cone) (red). Y-axis = % MT population. N = 10 neuronal cells. (TIF) [file pone.0097237.s002.tif]

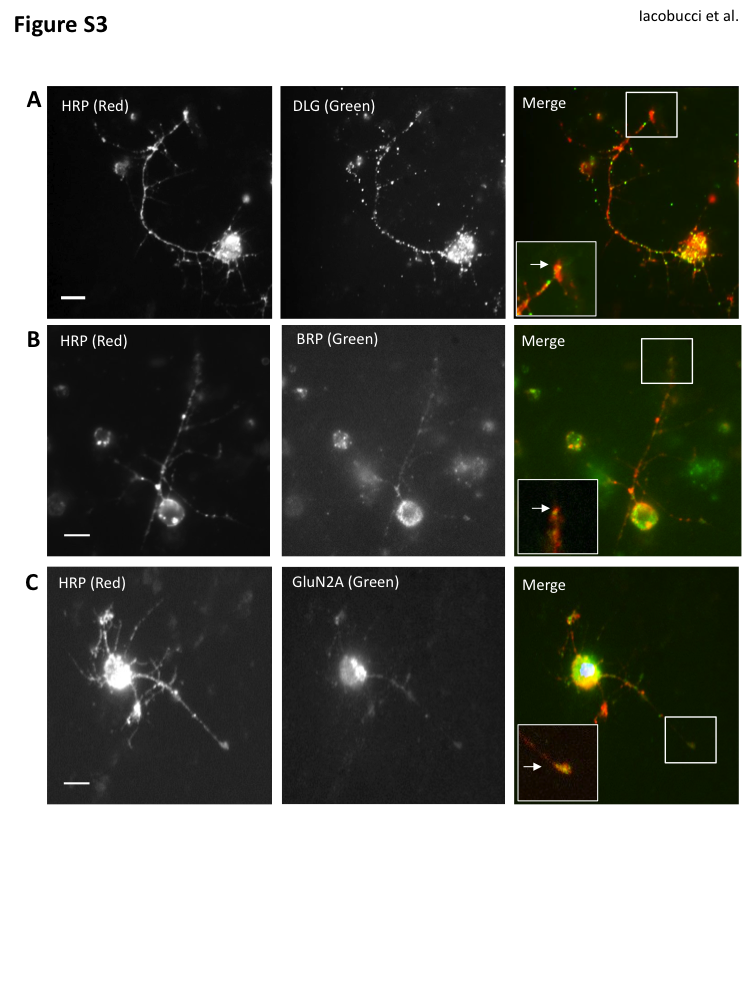

Supplement: Figure S3 — Neurons in primary culture contain proteins necessary to form functional synapses. (A) Two day old cultures show colocalization of HRP and DLG (discs large), at growth cones of neurites. Boxed region enlarged to show that both these proteins appear to co-localize into clusters at the growth cone (arrow). (B) Two day old neurons show Bruchpilot (BRP) in cell bodies and neurites. Arrow indicates co-localized clusters. (C) Two day old neurons show glutamate receptor subunit, GluN2A in the cell body and neurites. Arrow indicates co-localized clusters. Bar = 10 microns. (TIF) [file pone.0097237.s003.tif]

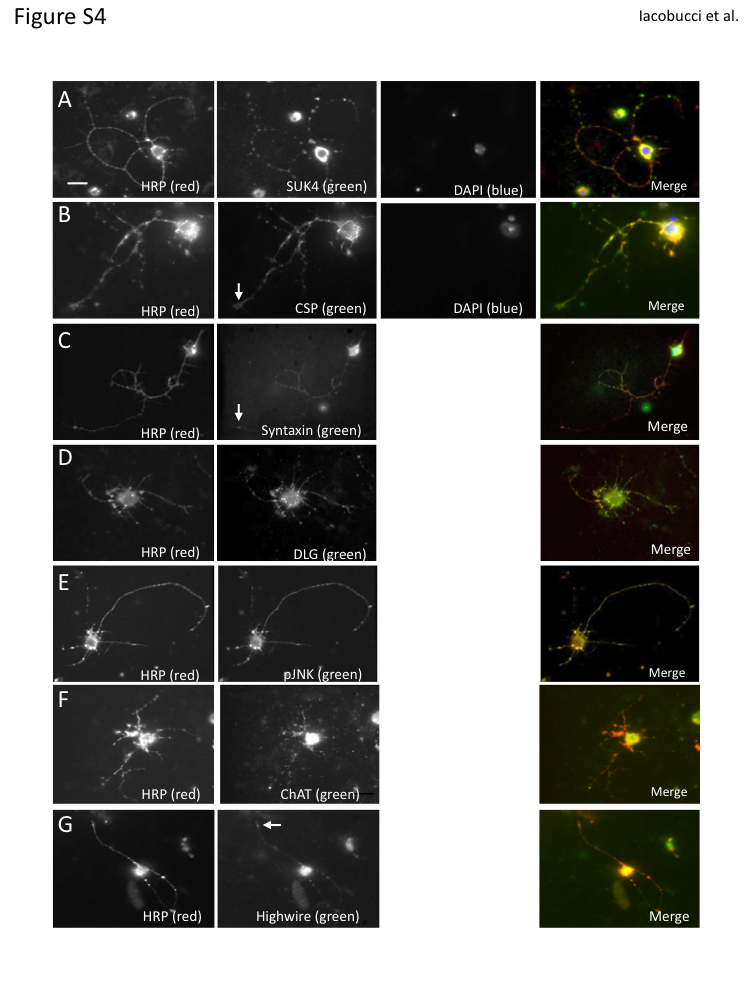

Supplement: Figure S4 — Primary neuronal cultures show cell bodies, axonal projections and growth cones as observed by several neuronal markers. Neuronal cultures were stained with several neuronal antibodies. All antibodies revealed strong localization in cell bodies. HRP was used as a neuronal marker. DAPI was used to reveal nuclei. (A) SUK4 showed uniform expression in neurite projections. (B) CSP was observed in all neurite projections. (C) Syntaxin showed strong staining at neurtie projections and growth cones (arrow). (D) DLG was found uniformly in all projections. (E) p-JNK was seen in all projections. (F) ChAT was found in the cell body only and was faintly seen in neurites and growth cones. (G) Highwire was strongly observed in the growth cone (arrow). Bar = 10 microns. (TIF) [file pone.0097237.s004.tif]

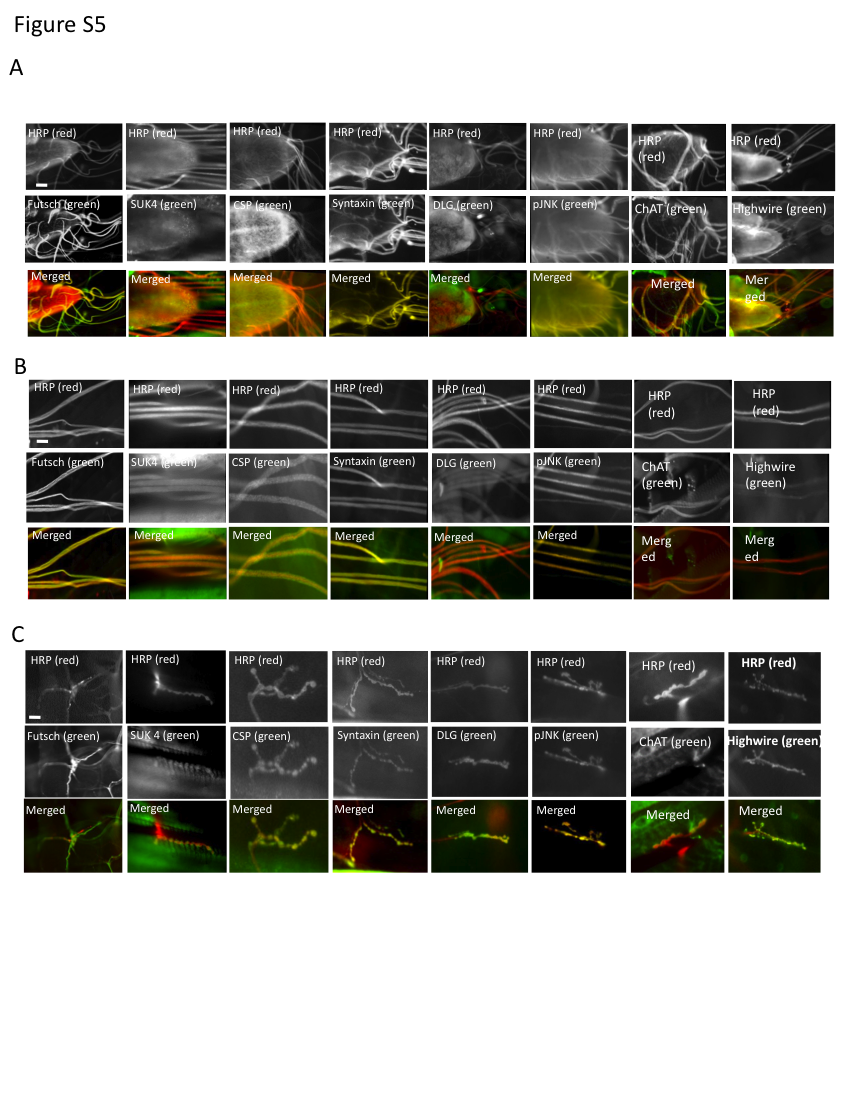

Supplement: Figure S5 — Localization of neuronal markers in larvae. (A) In the ventral ganglion, SUK 4, CSP, Syntaxin, DLG, p-JNK, and Highwire were observed. (B) While all antibodies were seen in larval segmental nerves, Futsch, SUK 4, CSP, Syntaxin, p-JNK, and ChAT were strongly observed in segmental nerves. (C) While all antibodies showed localization in neuromuscular junctions (NMJs), Futsch, CSP, Syntaxin, DLG, p-JNK, and Highwire were strongly observed in this anatomical structure. Bar = 10 microns. (TIF) [file pone.0097237.s005.tif]

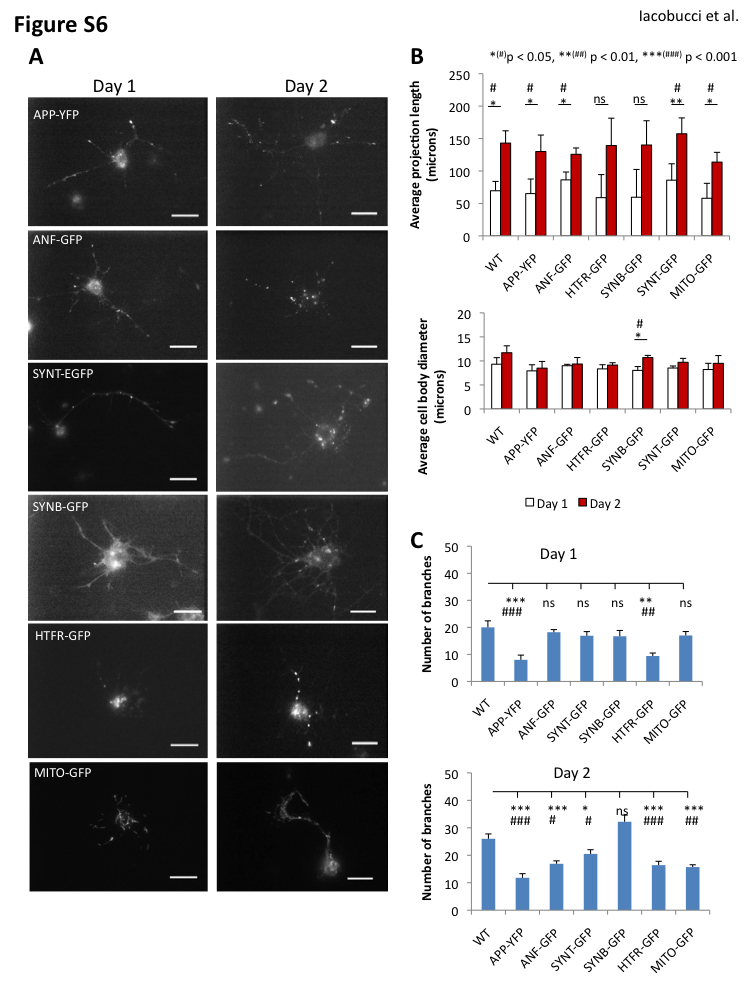

Supplement: Figure S6 — Expression of six GFP-tagged vesicles/organelle has no effect on neuronal growth over time. (A) Representative neuronal cells show neurite growth at day 2 relative to day 1 for all genotypes. (B) Quantification of cell body diameter and neurite length at day 1 and day 2 revealed increased rates of growth for each genotype, similar to neurites not expressing GFP/YFP tags. Only SYNB-GFP neurons showed a significant increase in cell body size (p = 0.041). (C) At day 1, APP-YFP (p = 9.03E-4) and HTFR-GFP (p = 0.002) neurons had significantly less branches than non-GFP/YFP expressing neurons. At day 2, only SYNB-GFP neurons had significantly more branches such that there was no significant difference compared to GFP/YFP non-expressing neurons. Bar = 10 microns. N = 10 cells; *(#) p<0.05, **(##) p<0.01, ***(###) p<0.001 by two-tailed Student t-test (*) and by Bonferroni’s test (#). NS = not significant. (TIF) [file pone.0097237.s006.tif]

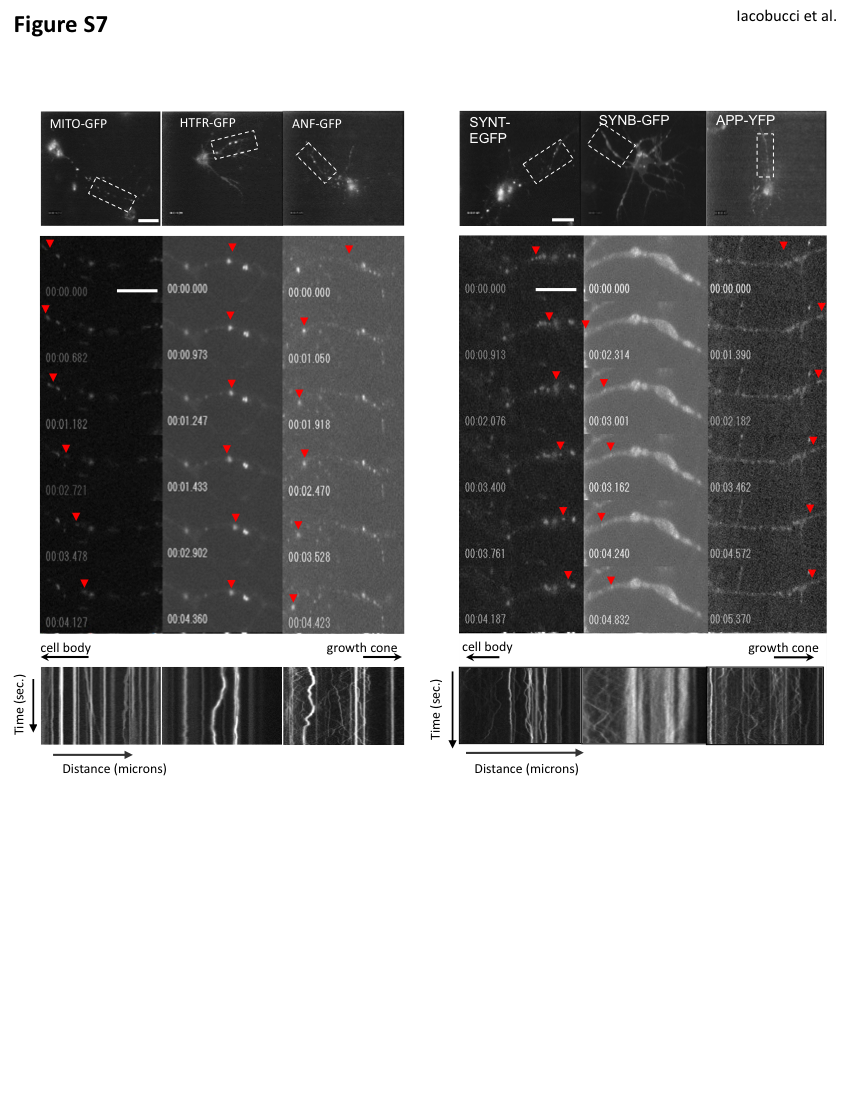

Supplement: Figure S7 — Robust bi-directional movement of GFP/YFP vesicles/organelle is observed in primary neurons. Representative movie montages show distinct moving vesicles/organelle for all genotypes. The boxed region is enlarged and shows the axonal neurite region used to generate the kymographs. Note that SYNB-GFP neurites show a characteristic diffused pattern in addition to discrete moving SYNB vesicles. Moving vesicle are depicted by arrowheads. Bar = 10 microns. (TIF) [file pone.0097237.s007.tif]

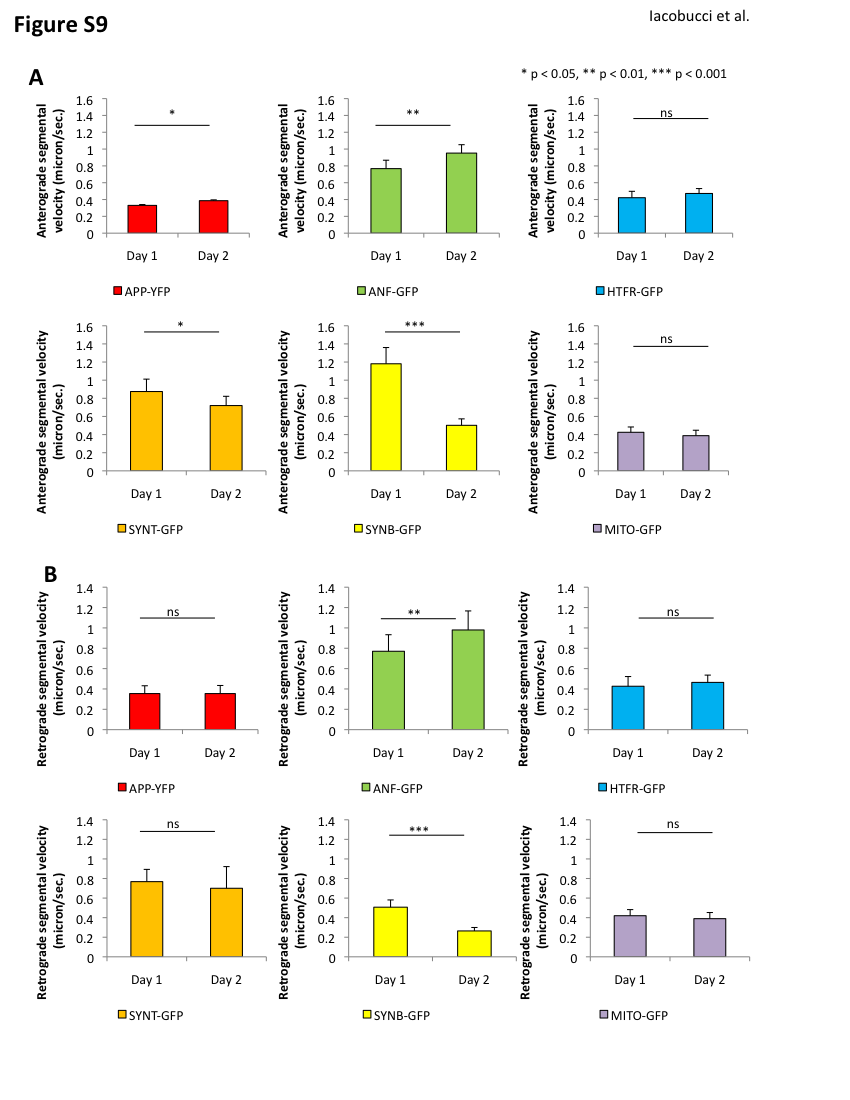

Supplement: Figure S9 — Anterograde and retrograde average segmental velocities of GFP/YFP tagged vesicles/cargo significantly change temporally. The average duration weighted segmental velocities of six vesicles/organelle revealed significant shifts in their movement dynamics at day 2 compared to day 1. (A) Consistent with analysis of duration weighted segmental velocities (Figure 2), the anterograde segmental velocity of APP-YFP vesicles significantly increased at day 2 (p = 0.046). Anterograde segmental velocity distributions of ANF-GFP vesicles also significantly increased at day 2 (p = 0.004). Anterograde segmental velocity of SYNB-GFP vesicles and SYNT-GFP vesicles significantly decreased over time (p = 1.658E-8 and p = 0.025, respectively). HTFR-GFP and MITO showed no significant change at day 2 compared to day 1. (B) The retrograde segmental velocity of ANF-GFP significantly increased (p = 0.008), while SYNB-GFP significantly decreased over time (p = 2.031E-4). APP-YFP, HTFR-GFP, SYNT-GFP, and MITO-GFP showed no significant changes at day 2 compared to day 1. N = 10 cells; *p<0.05, **p<0.01, ***p<0.001 by Wilcoxon-Mann-Whitney rank sum test for nonparametric distributions. NS = not significant. (TIF) [file pone.0097237.s009.tif]

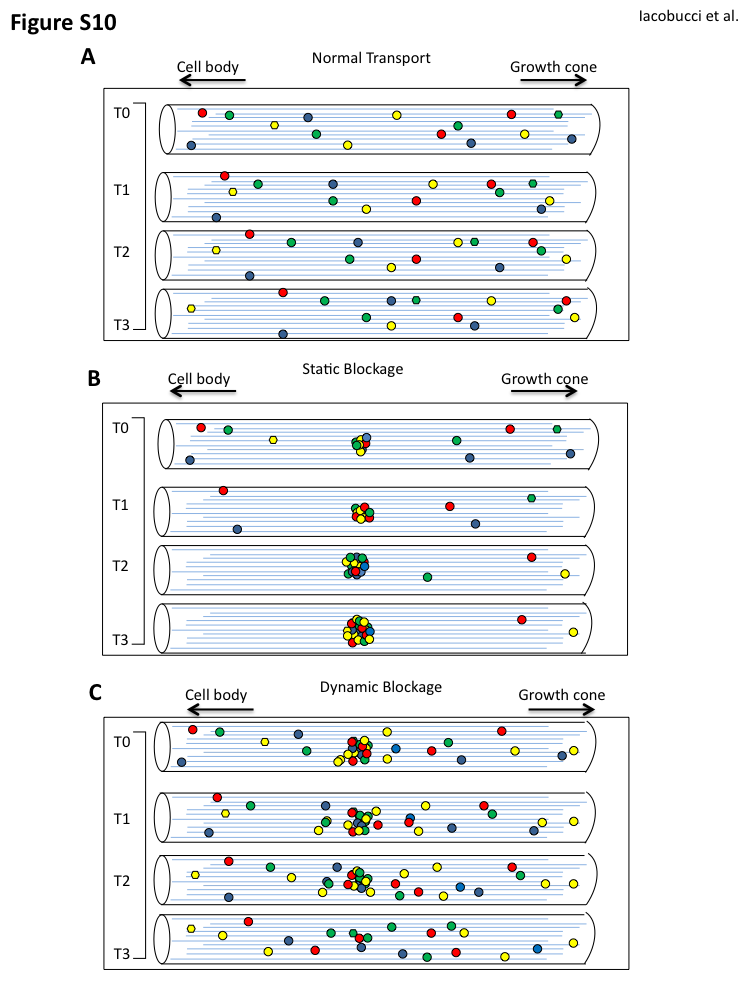

Supplement: Figure S10 — Proposed model for vesicle transport dynamics. (A) During development, bi-directional movement of vesicles over time (T0 to T3) is essential for the maintenance of the cell. (B) Aggregations of vesicles form and develop into blockages as they sequester other moving vesicles in the axon and directly impede transport. These blocks may likely cause detrimental defects to neuronal growth and function. (C) In normal development, transient blockages spontaneously form and resolve. These blocks do not cause a significant impairment to transport. These dynamic blocks may result due to subtle changes during transport and are likely benign. (TIF) [file pone.0097237.s010.tif]
