# Supplementary material for: Spatial and Temporal Characteristics of Normal and Perturbed Vesicle Transport
Source: PLoS One. 2014 May 30;9(5):e97237. doi: 10.1371/journal.pone.0097237 (PMC4039462; doi:10.1371/journal.pone.0097237)
Supplement: Table S1 — Summary of neuronal growth in primary neuronal cultures in GFP/YFP-expressing and non-expressing neurons. (DOC) [file pone.0097237.s011.doc]

Table S1: Summary of neuronal growth analysis

| **Genotype** | **Average Growth; mean +/- sem. (μm)** | | | | | | | | **Growth Rate Day 1 to Day 2 (μm/h)** |
| --- | --- | --- | --- | --- | --- | --- | --- | --- | --- |
|  |  | **Day 1** | **Day 2** | **P value**  **(Cohen’s D)** | **Day 3** | **P value**  **(Cohen’s D)** | **Day 4** | **P value**  **(Cohen’s D)** |  |
| **WT** | **Axon** | 69.54 +/- 14.36 | 143.36 +/- 18.96 | **0.016***  **0.041^#^**  (d = 4.39) | 186.75 +/- 22.46 | **0.042***  **0.049^#^**  (d = 2.09) | 189.1 +/- 27.64 | 0.132  0.444  (d = 0.093) | 3.08 |
|  | **Soma** | 9.31 +/- 1.37 | 11.70 +/- 1.45 | 0.186  0.999  (d = 1.70) | 12.45 +/- 2.3 | 0.235  0.654  (d = 0.39) | 13.13 +/- 2.09 | 0.187  0.879  (d = 0.31) | 0.10 |
| **APP-YFP** | **Axon** | 61.72 +/- 12.59 | 105.01 +/- 10.91 | **0.009****  **0.036^#^**  (d = 3.67) | n/a | n/a | n/a | n/a | 1.80 |
|  | **Soma** | 8.09 +/- 1.27 | 9.11 +/- 1.02 | 0.079  0.873  (d = 0.89) | n/a | n/a | n/a | n/a | 0.04 |
| **ANF-GFP** | **Axon** | 86.31 +/- 12.09 | 125.73 +/- 9.78 | **0.027***  **0.047^#^**  (d = 3.59) | 153.73 +/- 25.61 | **0.041***  0.087  (d = 1.45) | 158.70 +/- 35.64 | 0.246  0.989  (d = 0.16) | 1.64 |
|  | **Soma** | 9.00 +/- 0.29 | 9.35 +/- 1.36 | 0.458  0.246  (d = 0.36) | 9.4 +/- 4.5 | 0.532  0.889  (d = 0.02) | 14.8 +/- 1.08 | 0.169  0.475  (d = 1.65) | 0.02 |
| **SYNT-GFP** | **Axon** | 85.84 +/- 25.29 | 157.35 +/- 24.50 | **0.009****  **0.023^#^**  (d = 2.87) | n/a | n/a | n/a | n/a | 2.98 |
|  | **Soma** | 8.52 +/- 0.43 | 9.71 +/- 0.82 | 0.062  0.139  (d = 1.82) | n/a | n/a | n/a | n/a | 0.05 |
| **SYNB-GFP** | **Axon** | 59.54 +/- 42.85 | 140.04 +/- 37.44 | 0.191  0.212  (d = 2.00) | n/a | n/a | n/a | n/a | 3.35 |
|  | **Soma** | 8.03 +/- 0.80 | 10.68 +/- 0.47 | **0.034***  **0.049^#^**  (d = 4.04) | n/a | n/a | n/a | n/a | 0.11 |
| **HTFR-GFP** | **Axon** | 58.92 +/- 35.56 | 139.20 +/- 42.20 | 0.237  0.698  (d = 2.06) | n/a | n/a | n/a | n/a | 3.35 |
|  | **Soma** | 8.34 +/- 0.87 | 9.13 +/- 0.49 | 0.654  0.713  (d = 1.12) | n/a | n/a | n/a | n/a | 0.03 |
| **MITO-GFP** | **Axon** | 57.96 +/- 23.05 | 113.70 +/- 15.07 | **0.026***  **0.037^#^**  (d = 2.86) | n/a | n/a | n/a | n/a | 2.32 |
|  | **Soma** | 8.21 +/- 1.29 | 9.49 +/- 1.62 | 0.420  0.548  (d = 0.87) | n/a | n/a | n/a | n/a | 0.05 |

*Significance <0.05, **significance <0.01, ***significance <0.001 as determined by Student’s two-tailed t-test.

^#^Significance <0.05. ^##^Significance <0.01, ^###^Significance <0.001 as determined by Bonferroni test for multiple comparisons.

Effect size determined by Cohen’s D (d) as calculated by the mean difference and pooled standard deviation of two independent samples.
