# Supplementary material for: Spatial and Temporal Characteristics of Normal and Perturbed Vesicle Transport
Source: PLoS One. 2014 May 30;9(5):e97237. doi: 10.1371/journal.pone.0097237 (PMC4039462; doi:10.1371/journal.pone.0097237)
Supplement: Table S2 — Summary of GFP/YFP neurons in primary culture. (DOC) [file pone.0097237.s012.doc]

Table S2: Summary of GFP/YFP neurons

|  | **Day 1** | **Day 2** |
| --- | --- | --- |
| **Genotype** | **YFP/GFP expressing (mean +/- sem; %)** | **YFP/GFP expressing (mean +/- sem; %)** |
| **APP-YFP** | 45.7 +/- 6.9 | 51.7 +/- 7.1 |
| **ANF-GFP** | 43.2 +/- 7.2 | 64.5 +/- 10.2 |
| **SYNT-GFP** | 36.9 +/- 4.8 | 47.4 +/- 9.8 |
| **SYNB-GFP** | 32.7 +/- 3.6 | 66.7 +/- 9.6 |
| **HTFR-GFP** | 54.8 +/- 10.1 | 53.5 +/- 8.5 |
| **MITO-GFP** | 56.7 +/- 11.9 | 58.2 +/- 10.5 |
