# Supplementary material for: Spatial and Temporal Characteristics of Normal and Perturbed Vesicle Transport
Source: PLoS One. 2014 May 30;9(5):e97237. doi: 10.1371/journal.pone.0097237 (PMC4039462; doi:10.1371/journal.pone.0097237)
Supplement: Table S3 — Summary of neuronal branching in GFP/YFP-expressing and non-expressing neurons. (DOC) [file pone.0097237.s013.doc]

Table S3: Summary of neuronal branching analysis

|  | **Average branches per cell +/- sem.** | | **P-value of parameters compared to WT (Cohen’s D)** | |
| --- | --- | --- | --- | --- |
| **Genotype** | **Day 1** | **Day 2** | **Day 1** | **Day 2** |
| **WT** | 20.0 +/- 2.4 | 26.0 +/- 1.8 | n/a | n/a |
| **APP-YFP** | 8.0 +/- 1.7 | 11.8 +/- 1.5 | **7.08E-4*****  **7.16E-4^###^**  (d = 5.77) | **2.83E-4*****  **9.05E-7^###^**  (d = 8.57) |
| **ANF-GFP** | 18.2 +/- 0.9 | 16.9 +/- 1.1 | 0.202  1.000  (d = 0.99) | **2.63E-4*****  **0.010^#^**  (d = 6.10) |
| **SYNT-GFP** | 16.9 +/- 1.5 | 20.5 +/- 1.5 | 0.137  0.987  (d = 1.55) | **0.046***  **0.010^#^**  (d = 3.32) |
| **SYNB-GFP** | 16.7 +/- 2.2 | 32.2 +/- 2.4 | 0.321  0.977  (d = 1.43) | 0.085  0.303  (d = 2.92) |
| **HTFR-GFP** | 9.4 +/- 1.1 | 16.4 +/- 1.4 | **0.001****  **0.001^##^**  (d = 5.68) | **6.28E-4*****  **5.01E-9^###^**  (d = 5.95) |
| **MITO-GFP** | 17.0 +/- 1.5 | 15.7 +/- 0.8 | 0.243  0.990  (d = 1.50) | **2.13E-3*****  **0.001^##^**  (d = 7.40) |

*Significance <0.05, **significance <0.01, ***significance <0.001 as determined by Student’s two-tailed t-test.

^#^Significance <0.05. ^##^Significance <0.01, ^###^Significance <0.001 as determined by Bonferroni test for multiple comparisons.

Effect size determined by Cohen’s D (d) as calculated by the mean difference and pooled standard deviation of two independent samples.
