# Supplementary material for: Spatial and Temporal Characteristics of Normal and Perturbed Vesicle Transport
Source: PLoS One. 2014 May 30;9(5):e97237. doi: 10.1371/journal.pone.0097237 (PMC4039462; doi:10.1371/journal.pone.0097237)
Supplement: Table S4 — Summary of vesicle transport measurements in primary neuronal cultures using a custom single particle tracking software program. (DOC) [file pone.0097237.s014.doc]

**Table S4: Summary of vesicle/organelle motility measurements from axonal neurites using our** custom single particle tracking software program

|  | **APP-YFP** | | **ANF-GFP** | | **SYNT-GFP** | | **SYNB-GFP** | | **HTFR-GFP** | | **MITO-GFP** | |
| --- | --- | --- | --- | --- | --- | --- | --- | --- | --- | --- | --- | --- |
|  | **Day 1** | **Day 2** | **Day 1** | **Day 2** | **Day 1** | **Day 2** | **Day 1** | **Day 2** | **Day 1** | **Day 2** | **Day 1** | **Day 2** |
| Total number of vesicles | 505 | 514 | 266 | 319 | 202 | 292 | 202 | 239 | 136 | 152 | 177 | 188 |
| Stationary vesicles | 286  (56.6%) | 235  (45.7%)  P = 0.117  P=1.000 | 113  (42.5%) | 113  (35.4%)  P = 0.692  P=1.000 | 132  (65.3%) | 172  (58.9%)  P = 0.071  P=1.000 | 114  (56.4%) | 139  (58.2%)  P = 0.450  P=1.000 | 102  (75.0%) | 114  (75.0%)  P = 0.417  P=1.000 | 122  (68.9%) | 146  (77.7%)  P = 0.052  P=1.000 |
| Anterograde vesicles | 41  (8.1%) | 50  (9.7%)  P = 0.257  P=1.000 | 16  (6.0%) | 23  (7.2%)  P = 0.357  P=1.000 | 3  (1.5%) | 14  (4.8%) ↑  P = **0.009****  **P=0.010^#^** | 5  (2.5%) | 10  (4.2%)  P = 0.216  P=1.000 | 2  (1.5%) | 12  (7.9%) ↑  P = **0.010***  **P=0.044^#^** | 5  (2.8%) | 3  (1.6%)  P = 0.649  P=1.000 |
| Retrograde vesicles | 56  (11.1%) | 63  (12.3%)  P = 0.462  P=1.000 | 31  (11.7%) | 14  (4.4%) ↓  P = **0.018***  **P=0.032^#^** | 10  (4.9%) | 22  (7.5%)  P = 0.089  P=1.000 | 6  (2.9%) | 10  (4.2%)  P = 0.219  P=1.000 | 10  (7.4%) | 9  (5.9%)  P = 0.813  P=1.000 | 9  (5.1%) | 8  (4.3%)  P = 0.853  P=1.000 |
| Reversing vesicles | 122  (24.2%) | 166  (32.3%)  P = 0.072  P=1.000 | 106  (39.8%) | 169  (52.9%) ↑  P = **0.002****  **P=0.005^##^** | 57  (28.2%) | 84  (28.8%)  P = 0.562  P=1.000 | 77  (38.1%) | 80  (33.5%)  P = 0.377  P=1.000 | 22  (16.2%) | 17  (11.2%)  P = 0.444  P=1.000 | 41  (23.2%) | 31  (16.5%)  P = 0.697  P=1.000 |
| Anterograde duration weighted segmental velocity (mean±SEM; μm/sec.) | 0.331±0.083  N = 244 segments | 0.386±0.091  N = 336  ↑  P = **0.015***  (d = 0.63) | 0.768±0.073  N = 361 | 0.952±0.093  N = 420  ↑  P = **1.068E-6*****  (d = 2.18) | 0.875±0.069  N = 128 | 0.720±0.051  N = 259  ↓  P = **4.232E-4*****  (d = 2.69) | 0.591±0.089  N = 25 | 0.251±0.071  N = 232  ↓  P = **3.484E-6*****  (d = 4.67) | 0.422±0.076  N = 52 | 0.472±0.059  N = 37  P = 0.265  (d = 0.720) | 0.425±0.059  N = 99 | 0.388±0.060  N = 67  P = 0.211  (d = 0.623) |
| Retrograde duration-weighted segmental velocity (mean ±SEM; μm/sec.) | 0.355±0.077  N = 265 segments | 0.355±0.079  N = 368  P = 0.998  (d = 0.00) | 0.770±0.082  N = 422 | 0.980±0.094  N = 388  ↑  P = **1.092E-7*****  (d = 2.39) | 0.768±0.063  N = 204 | 0.700±0.111  N = 257  P = 0.054  (d = 0.732) | 0.507±0.074  N = 35 | 0.265±0.035  N = 241  ↓  P = **6.043E-7*****  (d = 5.78) | 0.427±0.096  N = 87 | 0.465±0.072  N = 49  P = 0.410  (d = 0.43) | 0.420±0.063  N = 111 | 0.389±0.063  N = 102  P = 0.269  (d = 0.49) |
| Anterograde segmental pause frequency (mean±SEM; pause/sec.) | 0.132±0.044  N = 114 Pauses | 0.075±0.006  N = 128  P = 0.625  P=0.999  (d = 1.87) | 0.077±0.006  N = 102 | 0.079±0.006  N = 122  P = 0.964  P=1.000  (d = 0.33) | 0.070±0.014  N = 26 | 0.111±0.014  N = 62  P = 0.093  P=0.142  (d = 2.93) | 0.064±0.029  N = 4 | 0.057±0.012  N = 30  P = 0.477  P=1.000  (d = 0.48) | 0.075±0.017  N = 11 | 0.049±0.009  N = 7  P = 0.147  P=0.989  (d = 1.79) | 0.066±0.010  N = 22 | 0.061±0.013  N = 12  P = 0.670  P=1.000  (d = 0.45) |
| Retrograde segmental pause frequency (mean ±SEM; pause/sec.) | 0.102±0.010  N = 115 Pauses | 0.170±0.057  N = 150  ↑  P = **0.014***  P=0.936  (d = 1.57) | 0.095±0.006  N = 124 | 0.072±0.006  N = 102  ↓  P = **0.007****  P=0.124  (d = 3.83) | 0.119±0.018  N = 37 | 0.110±0.012  N = 59  P = 0.466  P=0.992  (d = 0.62) | 0.094±0.059  N = 2 | 0.024±0.004  N = 35  P = 0.385  P=0.447  (d = 6.53) | 0.109±0.018  N = 18 | 0.062±0.011  N = 8  P = 0.154  P=0.929  (d = 2.89) | 0.078±0.016  N = 23 | 0.083±0.011  N = 25  P = 0.731  P=0.999  (d = 0.37) |
| Anterograde pause duration (mean ±SEM; sec.) | 0.368±0.024  N = 114 Pauses | 0.323±0.021  N = 128  P = 0.302  P=1.000  (d = 2.00) | 0.232±0.018  N = 156 | 0.236±0.017  N = 186  P = 0.847  P=1.000  (d = 0.23) | 0.131±0.022  N = 42 | 0.198±0.023  N = 133  P = 0.298  P=1.000  (d = 2.94) | 0.230±0.183  N = 4 | 0.186±0.032  N = 67  P = 0.752  P=1.000  (d = 0.89) | 0.128±0.031  N = 20 | 0.097±0.032  N = 9  P = 0.760  P=1.000  (d = 0.99) | 0.215±0.043  N = 29 | 0.152±0.034  N = 17  P = 0.092  P=1.000  (d = 1.58) |
| Retrograde pause duration (mean ± SEM; sec.) | 0.388±0.026  N = 115 Pauses | 0.339±0.021  N = 150  P = 0.874  P=1.000  (d = 2.10) | 0.258±0.017  N = 225 | 0.211±0.016  N = 152  P = 0.961  P=1.000  (d = 2.83) | 0.205±0.025  N = 89 | 0.229±0.022  N = 128  ↑  P = **8.340E-4*****  P=1.000  (d = 1.03) | 0.197±0.150  N = 4 | 0.126±0.026  N = 39  ↓  P = **0.034***  P=1.000  (d = 1.49) | 0.224±0.038  N = 40 | 0.097±0.019  N = 13  ↓  P = **0.026***  P=1.000  (d = 3.68) | 0.169±0.032  N = 38 | 0.205±0.032  N = 44  P = 0.443  P=1.000  (d = 1.13) |

*Significance <0.05, **significance <0.01, ***significance <0.001.

Significance of cargo population determined by Student’s two-tailed t-test.

Significance of duration-weighted segmental velocity determined by Wilcoxon-Mann-Whitney rank sum test after Andersen-Darling test confirmed this data followed non normal distributions.

Significance of segmental pause frequency determined by Student’s two-tailed t-test.

Significance of pause duration determined by Student’s two-tailed t-test.

^#^Significance <0.05. ^##^Significance <0.01, ^###^Significance <0.001 as determined by Bonferroni test for multiple comparisons.

Effect size determined by Cohen’s D (d) as calculated by the mean difference and pooled standard deviation of two independent samples.
