# Supplementary material for: Spatial and Temporal Characteristics of Normal and Perturbed Vesicle Transport
Source: PLoS One. 2014 May 30;9(5):e97237. doi: 10.1371/journal.pone.0097237 (PMC4039462; doi:10.1371/journal.pone.0097237)
Supplement: Table S5 — Summary of vesicle/organelle motility measurements in larvae using the custom single particle tracking software program. (DOC) [file pone.0097237.s015.doc]

**Table S5: Summary of *in vivo* vesicle/organelle motility measurements from larval axons using our custom single particle tracking software program**

|  | **APP-YFP** | **ANF-GFP** | **SYNT-GFP** | **SYNB-GFP** | **HTFR-GFP** | **MITO-GFP** |
| --- | --- | --- | --- | --- | --- | --- |
| **Total number of vesicles** | 250 | 216 | n/a | 207 | 196 | 208 |
| **Stationary vesicles** | 16  (6.40%) | 30  (13.9%) | n/a | 97  (46.9%) | 105  (53.6%) | 101  (48.6%) |
| **Anterograde vesicles** | 31  (12.4%) | 26  (12.0%) | n/a | 40  (19.3%) | 44  (22.4%) | 32  (15.4%) |
| **Retrograde vesicles** | 39  (15.6%) | 41  (19.0%) | n/a | 20  (9.7%) | 8  (4.1%) | 31  (14.9%) |
| **Reversing vesicles** | 164  (65.6%) | 119  (55.1%) | n/a | 50  (24.2%) | 39  (19.9%) | 44  (21.2%) |
| **Anterograde duration weighted segmental velocity (mean±SEM; μm/sec.)** | 0.494±0.104  N = 209 segments | 0.889±0.218  N = 240 | n/a | 0.251±0.148  N = 149 | 0.479±0.121  N = 155 | 0.519±0.067  N = 120 |
| **Retrograde duration-weighted segmental velocity (mean ±SEM; μm/sec.)** | 0.492±0.110  N = 208 segments | 0.874±0.215  N = 264 | n/a | 0.264±0.139  N = 114 | 0.516±0.161  N = 94 | 0.458±0.060  N = 123 |
| **Anterograde segmental pause frequency (mean±SEM; pause/sec.)** | 0.040±0.009  N = 56 pauses | 0.050±0.005  N = 69 | n/a | 0.011±0.004  N = 59 | 0.187±0.088  N = 51 | 0.034±0.005  N = 44 |
| **Retrograde segmental pause frequency (mean ±SEM; pause/sec.)** | 0.046±0.014  N = 58 pauses | 0.047±0.007  N = 83 | n/a | 0.012±0.010  N = 38 | 0.129±0.035  N = 64 | 0.044±0.009  N = 44 |
| **Anterograde pause duration (mean ±SEM; sec.)** | 0.046±0.004  N = 67 pauses | 0.158±0.020  N = 75 | n/a | 0.341±0.227  N = 52 | 0.308±0.037  N = 67 | 0.162±0.031  N = 30 |
| **Retrograde pause duration (mean ± SEM; sec.)** | 0.063±0.008  N = 80 pauses | 0.147±0.019  N = 77 | n/a | 0.256±0.035  N = 57 | 0.227±0.046  N = 71 | 0.158±0.031  N = 34 |
