# Supplementary material for: Spatial and Temporal Characteristics of Normal and Perturbed Vesicle Transport
Source: PLoS One. 2014 May 30;9(5):e97237. doi: 10.1371/journal.pone.0097237 (PMC4039462; doi:10.1371/journal.pone.0097237)
Supplement: Table S6 — Summary of neuronal growth measurements with 50% motor protein reduction in primary neuronal cultures. (DOC) [file pone.0097237.s016.doc]

Table S6: Summary of neuronal growth with 50% reduction of motor proteins

| **Genotype** | **Average Growth (μm)** | | | | | | | | **Growth Rate (μm/h)** | |
| --- | --- | --- | --- | --- | --- | --- | --- | --- | --- | --- |
|  | **Day 1** | | | | **Day 2** | | | |  | |
|  | **Axon** | **p-value compared to APP-YFP (Cohen’s D)** | **Soma** | **p-value compared to APP-YFP (Cohen’s D)** | **Axon** | **p-value compared to APP-YFP**  **(Cohen’s D)** | **Soma** | **p-value compared to APP-YFP**  **(Cohen’s D)** | **Axon** | **Soma** |
| **APP-YFP** | 61.72 +/- 12.59 | n/a | 8.09 +/- 1.27 | n/a | 105.01 +/- 10.91 | n/a | 9.11 +/- 1.02 | n/a | 1.80 | 0.04 |
| **APP-YFP; khc^20^ -/+** | 58.21 +/- 14.18 | 0.531  0.752  (d = 0.26) | 7.49 +/- 1.72 | 0.710  1.000  (d = 0.40) | 78.55 +/- 11.62 | **0.021***  **0.039^#^**  (d = 2.35) | 8.5 +/- 2.56 | 0.883  0.901  (d = 0.31) | 0.85 | 0.04 |
| **APP-YFP; robl^k^ -/+** | 65.72 +/- 25.85 | 0.823  0.999  (d = 0.20) | 5.24 +/- 0.37 | **0.034***  **0.040^#^**  (d = 3.05) | 80.65 +/- 9.86 | **0.015***  **0.042^#^**  (d = 2.34) | 6.17 +/- 0.29 | **0.026***  **0.043^#^**  (d = 3.92) | 0.62 | 0.03 |

*Significance <0.05, **significance <0.01, ***significance <0.001 as determined by Student’s two-tailed t-test.

^#^Significance <0.05. ^##^Significance <0.01, ^###^Significance <0.001 as determined by Bonferroni test for multiple comparisons.

Effect size determined by Cohen’s D (d) as calculated by the mean difference and pooled standard deviation of two independent samples.
