# Supplementary material for: Spatial and Temporal Characteristics of Normal and Perturbed Vesicle Transport
Source: PLoS One. 2014 May 30;9(5):e97237. doi: 10.1371/journal.pone.0097237 (PMC4039462; doi:10.1371/journal.pone.0097237)
Supplement: Table S7 — Summary of all vesicle transport measurements with 50% motor protein reduction in primary neuronal cultures. (DOC) [file pone.0097237.s017.doc]

**Table S7: Summary vesicle motility with 50% reduction of motor proteins**

|  | APP-YFP (200 msec control) | | APP-YFP; khc^20^ /+ (200 msec) | | APP-YFP (1000 msec control) | | APP-YFP; robl^K^ -/+ (1000 msec) | |
| --- | --- | --- | --- | --- | --- | --- | --- | --- |
|  | Day 1 | Day 2 | Day 1 | Day 2 | Day 1 | Day 2 | Day 1 | Day 2 |
| Total number of vesicles | 278 | 537 | 328 | 562 | 256 | 133 | 256 | 189 |
| Stationary vesicles | 135  (48.56%) | 246  (45.81%) | 196  (59.76%)  ↑  P = **0.002****  P = **0.033^#^** | 321  (57.12%)  ↑  P = **0.031***  **P = 0.044^#^** | 56  (21.88%) | 22  (16.54%) | 155  (60.55%)  ↑  P = **0.003****  **P = 0.011^#^** | 113  (59.79%)  ↑  P = **0.002****  **P = 0.021^#^** |
| Anterograde vesicles | 11  (3.96%) | 53  (9.87%) | 25  (7.62%)  P = 0.057  P = 0.110 | 57  (10.14%)  P = 0.156  P = 0.222 | 12  (4.69%) | 19  (14.29%) | 23  (8.98%)  P = 0.061  P = 0.389 | 8  (4.23%)  P = 0.083  P = 0.999 |
| Retrograde vesicles | 23  (8.27%) | 63  (11.73%) | 36  (10.98%)  P = 0.089  P = 0.094 | 69  (12.28%)  P = 0.097  P = 0.135 | 14  (5.47%) | 5  (3.76%) | 11  (4.30%)  P = 0.231  P = 1.000 | 5  (2.65%)  P = 0.185  P = 1.000 |
| Reversing vesicles | 109  (39.21%) | 175  (32.59%) | 71  (21.64%)  ↓  P = **0.013****  P = 0.063 | 115  (20.46%)  ↓  P = **0.021***  **P = 0.047^#^** | 174  (67.97%) | 87  (65.41%) | 67  (26.17%)  ↓  P = **0.004****  **P = 0.010^#^** | 63  (33.33%)  ↓  P = **0.002****  **P = 0.039^#^** |
| Anterograde duration weighted segmental velocity (mean±SEM; μm/sec.) | 0.336±0.087  N = 244  segments | 0.386±0.091  N = 335 | 0.257±0.054  N = 193  ↓  P = **2.514E-4*****  (d = 1.06) | 0.345±0.077  N = 337  ↓  P = **0.0454***  (d = 0.49) | 0.060±0.014  N = 493 | 0.065±0.017  N = 286 | 0.044±0.011  N = 202  ↓  P = **1.814E-6*****  (d = 1.21) | 0.053±0.010  N = 187  ↓  P = **0.005****  (d = 0.82) |
| Retrograde duration-weighted segmental velocity (mean ±SEM; μm/sec.) | 0.355±0.077  N = 264 segments | 0.355±0.080  N = 367 | 0.273±0.059  N = 196  ↓  P = **5.523E-5*****  (d = 1.17) | 0.319±0.073  N = 353  ↓  P = **0.044***  (d = 0.47) | 0.059±0.051  N = 521 | 0.074±0.021  N = 255 | 0.049±0.011  N = 162  ↓  P = **0.005****  (d = 0.22) | 0.054±0.011  N = 170  ↓  P = **6.743E-5*****  (d = 1.13) |
| Anterograde segmental pause frequency (mean±SEM; pause/sec.) | 0.132±0.044  N = 140  Pauses | 0.075±0.006  N = 152 | 0.156±0.077  N = 81  P = 0.234  P=1.000  (d = 0.38) | 0.222±0.076  N = 111  ↑  P = **0.034***  **P=0.048^#^**  (d = 2.97) | 0.013±0.001  N = 77 | 0.014±0.002  N = 60 | 0.022±0.003  N = 57  ↑  P = **0.021***  **P=0.033^#^**  (d = 4.29) | 0.021±0.004  N = 33  ↑  P = **0.042***  **P=0.044^#^**  (d = 2.44) |
| Retrograde segmental pause frequency (mean ±SEM; pause/sec.) | 0.102±0.010  N = 144  Pauses | 0.170±0.057  N = 162 | 0.157±0.066  N = 101  P = 0.341  P=0.999  (d = 1.28) | 0.463±0.116  N = 123  ↑  P = **0.002****  **P=0.011^#^**  (d = 3.35) | 0.013±0.001  N = 84 | 0.012±0.001  N = 41 | 0.016±0.002  N = 38  P = 0.062  P=1.000  (d = 2.16) | 0.014±0.001  N = 38  P = 0.079  P=1.000  (d = 2.00) |
| Anterograde pause duration (mean ±SEM; sec.) | 0.368±0.024  N = 140  Pauses | 0.323±0.021  N = 152 | 0.345±0.029  N = 81  P = 0.635  P=1.000  (d = 0.89) | 0.424±0.025  N = 111  ↑  P = **0.028***  **P=0.001^##^**  (d = 4.44) | 0.183±0.020  N = 110 | 0.228±0.028  N = 80 | 0.342±0.031  N = 87  ↑  P = **4.284E-5*****  **P=3.26E-13^###^**  (d = 6.25) | 0.269±0.044  N = 49  P = 0.234  P=0.999  (d = 1.17) |
| Retrograde pause duration (mean ± SEM; sec.) | 0.388±0.026  N = 144  Pauses | 0.424±0.025  N = 162 | 0.392±0.029  N = 101  P = 0.765  P=1.000  (d = 0.15) | 0.451±0.027  N = 123  ↑  P = **0.039***  **P= 0.040^#^**  (d = 1.04) | 0.184±0.018  N = 116 | 0.188±0.027  N = 55 | 0.283±0.041  N = 50  ↑  P = **0.015****  **P=3.61E-8^###^**  (d = 3.67) | 0.278±0.038  N = 51  ↑  P = **0.043***  **P=8.04E-4^###^**  (d = 2.75) |

*Significance <0.05, **significance <0.01, ***significance <0.001.

Significance of cargo population determined by Student’s two-tailed t-test.

Significance of duration-weighted segmental velocity determined by Wilcoxon-Mann-Whitney rank sum test after Andersen-Darling test confirmed this data followed non normal distributions.

Significance of segmental pause frequency determined by Student’s two-tailed t-test.

Significance of pause duration determined by Student’s two-tailed t-test.

^#^Significance <0.05. ^##^Significance <0.01, ^###^Significance <0.001 as determined by Bonferroni test for multiple comparisons.

Effect size determined by Cohen’s D (d) as calculated by the mean difference and pooled standard deviation of two independent samples.
