# Supplementary material for: Spatial and Temporal Characteristics of Normal and Perturbed Vesicle Transport
Source: PLoS One. 2014 May 30;9(5):e97237. doi: 10.1371/journal.pone.0097237 (PMC4039462; doi:10.1371/journal.pone.0097237)
Supplement: Table S8 — Summary of blockage characterization. (DOC) [file pone.0097237.s018.doc]

Table S8: Summary of axonal blockage analysis

| Genotype | Day 1 | | | | | | | Day 2 | | | | | | |
| --- | --- | --- | --- | --- | --- | --- | --- | --- | --- | --- | --- | --- | --- | --- |
|  | # static blocks | Percent static blocks (%) | P value  (Cohen’s D) | # dynamic blocks | Percent dynamic blocks (%) | P value  (Cohen’s D) | Normalized static/ dynamic block ratio | # static blocks | Percent static blocks (%) | P value  (Cohen’s D) | # dynamic blocks | Percent dynamic blocks (%) | P value  (Cohen’s D) | Normalized static/ dynamic block ratio |
| APP-YFP (APP-YFP; khc^20^ -/+ control) | 11 | 35.3 +/- 9.6 | **0.002****  **2.11E-4^###^**  (d = 5.94) | 19 | 54.6 +/- 10.7 | **0.019***  **0.002^##^**  (d = 3.35) | 1.00 | 19 | 51.5 +/- 7.1 | **0.005****  **0.032^#^**  (d = 4.75) | 15 | 38.5 +/- 6.05 | **0.005****  **0.012^#^**  (d = 3.14) | 2.07 |
| APP-YFP; khc^20^ -/+ | 31 | 77.7 +/- 6.1 |  | 8 | 22.3 +/- 6.1 |  | 5.38 | 26 | 79.2 +/- 4.7 |  | 8 | 20.8 +/- 4.7 |  | 5.88 |
| APP-YFP (APP-YFP; robl^K^ -/+ control) | 10 | 30.2 +/- 6.0 | **0.004****  **0.045^#^**  (d = 4.12) | 26 | 69.8 +/- 6.0 | **0.043***  **0.043^#^**  (d = 5.21) | 1.00 | 25 | 48.9 +/- 6.2 | **0.043***  **0.039^#^**  (d = 3.11) | 28 | 51.0 +/- 6.2 | **0.043***  **0.044^#^**  (d = 3.23) | 2.22 |
| APP-YFP; robl^K^ -/+ | 27 | 68.5 +/- 10.2 |  | 10 | 31.5 +/- 10.2 |  | 5.03 | 27 | 70.2 +/- 7.4 |  | 12 | 29.8 +/- 7.4 |  | 5.44 |

*Significance <0.05, **significance <0.01, ***significance <0.001 as determined by Student’s two-tailed t-test.

^#^Significance <0.05. ^##^Significance <0.01, ^###^Significance <0.001 as determined by Bonferroni test for multiple comparisons.

All static/dynamic block ratios are normalized to APP-YFP day 1.

Effect size determined by Cohen’s D (d) as calculated by the mean difference and pooled standard deviation of two independent samples.
