# Supplementary material for: Spatial and Temporal Characteristics of Normal and Perturbed Vesicle Transport
Source: PLoS One. 2014 May 30;9(5):e97237. doi: 10.1371/journal.pone.0097237 (PMC4039462; doi:10.1371/journal.pone.0097237)
Supplement: Table S9 — Summary of temporal predictor analysis of blockage type using logistic regression analysis. (DOC) [file pone.0097237.s019.doc]

**Table S9: Summary of logistic regression analysis of temporal changes in axonal** blockage type

| Analysis Type | Dependent variable | Variable selected | Odds Ratio (OR) | 95% Confidence Interval (CI) | β | p | % Correctly classified |
| --- | --- | --- | --- | --- | --- | --- | --- |
| Between-genotypes at the same timepoint | Probability of static blocks in APP-YFP (day 1) | Probability of static blocks in APP-YFP; khc^20^ -/+ (day 1) | 6.69 | 2.28-19.59 | 1.90 | **2.3E-3***** | 63.4 |
|  | Probability of static blocks in APP-YFP (day 2) | Probability of static blocks in APP-YFP; khc^20^ -/+ (day 2) | 2.57 | 0.91-7.29 | 0.94 | **0.001**** | 60.3 |
|  | Probability of static blocks in APP-YFP (day 1) | Probability of static blocks in APP-YFP; robl^K^ -/+ (day 1) | 7.02 | 2.66-17.29 | 1.95 | **2.3E-3***** | 72.6 |
|  | Probability of static blocks in APP-YFP (day 2) | Probability of static blocks in APP-YFP; robl^K^ -/+ (day 2) | 2.52 | 0.98-5.03 | 0.92 | **0.001**** | 59.8 |
| Within-genotypes over time | Probability of static blocks in APP-YFP (APP-YFP; khc^20^ -/+ control, day 2) | Probability of static blocks in APP-YFP (APP-YFP; khc^20^ -/+ control, day 1) | 2.19 | 0.92-3.96 | 0.78 | **0.015*** | 59.4 |
|  | Probability of static blocks in APP-YFP (APP-YFP; robl^K^ -/+ control, day 2) | Probability of static blocks in APP-YFP (APP-YFP; robl^K^ -/+ control, day 1) | 2.32 | 1.09-4.21 | 0.84 | **0.001**** | 46.6 |
|  | Probability of static blocks in APP-YFP; khc^20^ -/+ (day 2) | Probability of static blocks in APP-YFP; khc^20^ -/+ (day 1) | 1.09 | 0.76-1.87 | 0.09 | 0.493 | 57.3 |
|  | Probability of static blocks in APP-YFP; robl^K^ -/+ (day 2) | Probability of static blocks in APP-YFP; khc^20^ -/+ (day 1) | 1.08 | 0.68-1.23 | 0.08 | 0.413 | 48.7 |

*Significance <0.05, **significance <0.01, ***significance <0.001. Significance determined by Fisher’s exact t-test. Logistic regression analysis uses odds to model a binary dependent categorical variable (eg. Blockage type: static or dynamic) using one or more categorical or non-categorical variables (eg. Time: day 1 or day 2; genotype). Odds ratio (OR) is a measure of effect size by measuring the ratio of the odds that an event or result will occur to the odds of the event not occurring. Confidence interval (CI) is a measure of test precision that represents the range of odds ratios in which there can likely be a significant effect. Using 95% as the confidence coefficient, this interval gives us the result of testing the null hypothesis that the odds ratio is 1 for a signiﬁcance level of 0.05. If the conﬁdence interval does not include 1, it is more likely that there is a significant effect. The β value is the natural log of the odds ratio. This value demonstrates the directionality of the effect: positive values indicate the effect is in a positive direction while negative values would indicate a negative relationship. The % correctly classified value is a measure of test accuracy. This value reports the percentage of cases (blockages) that followed the predicted outcome (in our case, the percentage of blockages correctly predicted to increase in static blocks at day 2 out of the three possible blockage outcomes: no change, increased static blocks, or decreased static blocks).
